# Amino acid starvation sensing dampens IL-1β production by activating riboclustering and autophagy

**DOI:** 10.1371/journal.pbio.2005317

**Published:** 2018-04-05

**Authors:** Srikanth Battu, Sumbul Afroz, Jeevan Giddaluru, Saima Naz, Weishan Huang, Saratchandra Singh Khumukcham, Rafiq Ahmad Khan, Saleem Yousuf Bhat, Insaf Ahmed Qureshi, Bramanandam Manavathi, Aleem Ahmed Khan, Avery August, Seyed Ehtesham Hasnain, Nooruddin Khan

**Affiliations:** 1 Department of Biotechnology and Bioinformatics, School of Life Sciences, University of Hyderabad, Hyderabad, Telangana, India; 2 Centre for Liver Research and Diagnostics, Central Laboratory for Stem Cell Research and Translational Medicine, Deccan College of Medical Sciences, Kanchanbagh, Hyderabad, Telangana, India; 3 Department of Microbiology and Immunology, College of Veterinary Medicine, Cornell University, Ithaca, New York, United States of America; 4 Department of Biochemistry, School of Life Sciences, University of Hyderabad, Hyderabad, Telangana, India; 5 JH-Institute of Molecular Medicine, Jamia Hamdard University, Hamdard Nagar, New Delhi, India; 6 Molecular Infection and Functional Biology Laboratory, Kusuma School of Biological Sciences, Indian Institute of Technology, New Delhi, India; 7 Dr Reddy’s Institute of Life Sciences, University of Hyderabad Campus, Hyderabad, Telangana, India; St. Jude Childrens Research Hospital, Memphis, United States of America

## Abstract

Activation of the amino acid starvation response (AAR) increases lifespan and acute stress resistance as well as regulates inflammation. However, the underlying mechanisms remain unclear. Here, we show that activation of AAR pharmacologically by Halofuginone (HF) significantly inhibits production of the proinflammatory cytokine interleukin 1β (IL-1β) and provides protection from intestinal inflammation in mice. HF inhibits IL-1β through general control nonderepressible 2 kinase (GCN2)–dependent activation of the cytoprotective integrated stress response (ISR) pathway, resulting in rerouting of IL-1β mRNA from translationally active polysomes to inactive ribocluster complexes—such as stress granules (SGs)—via recruitment of RNA-binding proteins (RBPs) T cell–restricted intracellular antigen-1(TIA-1)/TIA-1–related (TIAR), which are further cleared through induction of autophagy. GCN2 ablation resulted in reduced autophagy and SG formation, which is inversely correlated with IL-1β production. Furthermore, HF diminishes inflammasome activation through suppression of reactive oxygen species (ROS) production. Our study unveils a novel mechanism by which IL-1β is regulated by AAR and further suggests that administration of HF might offer an effective therapeutic intervention against inflammatory diseases.

## Author summary

Reduced intake of food (also known as dietary restriction) without malnutrition has been shown to benefit health in humans and animals, including an increase in life expectancy, metabolic fitness, and resistance to acute stress. Recent studies have attributed the benefits associated with dietary restriction to the reduced intake of amino acids. However, the underlying mechanisms through which amino acid restriction regulates various homeostatic processes are poorly defined. Here, we show that activation of amino acid starvation response (AAR) by the small molecule Halofuginone (HF) results in a significant inhibition of production of interleukin 1β (IL-1β), a proinflammatory mediator. We find that AAR provides protection from intestinal inflammation–associated pathology in a mouse model of colitis through a novel mechanism involving the formation of riboclusters (groups of RNA-binding proteins (RBPs) and stalled mRNA complexes) and autophagy. We further show that HF-mediated inhibition in IL-1β production is dependent on general control nonderepressible 2 kinase (GCN2), an amino acid deprivation sensor. This study provides the mechanisms regulating AAR-induced benefits in the context of inflammation and further suggests that the administration of HF might offer an effective therapeutic intervention against inflammatory diseases in mammals.

## Introduction

Dietary amino acid restriction, without malnutrition, offers enormous health benefits, including longevity of lifespan [1], acute stress resistance, increased insulin sensitivity, and modulation of inflammation [2,3]. However, the underlying mechanisms through which amino acid restriction extends its beneficial effects remain poorly defined. General control nonderepressible 2 kinase (GCN2) is a well-known metabolic sensor, which senses amino acid starvation conditions and programs protein synthesis through activation of the homeostatic integrated stress response (ISR) [4]. The ISR is an evolutionarily conserved homeostatic process that enables mammalian cells to sense, adapt, and appropriately respond to a wide variety of extracellular and intracellular stress signals. Four distinct eukaryotic initiation factor 2 (eIF2) kinases—including GCN2, RNA-dependent protein kinase-like endoplasmic reticulum kinase (PERK), protein kinase R (PKR), and heme-regulated eIF2α kinase (HRI)—mediate the ISR [5]. GCN2 senses amino acid insufficiency, PERK is activated by endoplasmic reticulum stress, PKR senses viral double-stranded RNA (dsRNA), and HRI senses heme deprivation, respectively [6].

Depletion of intracellular amino acids results in the accumulation of uncharged transfer RNAs (tRNAs) that bind to GCN2 [6], leading to a conformational change and kinase activation. Phosphorylated GCN2, in turn, triggers inhibitory phosphorylation of eIF2α, a crucial eukaryotic translation initiation factor, resulting in impaired assembly of eIF2-guanosine triphosphate (GTP)-tRNA^Met^ and polysome formation [5]. This represses translational initiation and protein synthesis in cells to economize energy and adapt to the conditions of amino acid starvation. GCN2-mediated translational blockade results in the accumulation of translationally silenced mRNAs, which further undergo various post-transcriptional reprogramming (PTR) via recruitment of RNA-binding proteins (RBPs), leading to the formation of RBP–RNA complexes known as “riboclusters” such as stress granules (SGs). In SGs, the RBP–RNA composition determines the fate of mRNAs translatability, decay, or its storage [7].

In addition to GCN2, mammalian target of rapamycin (mTOR), a serine/threonine kinase, directly senses amino acid availability in the cytosol via an unknown mechanism. mTOR orchestrates anabolic processes such as fatty acid synthesis and cell growth by integrating the supply of energy, nutrients, and growth factors [8]. Pharmacological inhibition of mTOR signaling also increases lifespan, suggesting cross-talk between GCN2 and mTOR signaling pathways [8]. Halofuginone (HF), a mimetic of the amino acid starvation response (AAR), is a small molecule derived from the plant alkaloid febrifugine, extracted from the herb *Dichroa febrifuga*, and has gained significant attention for its therapeutic value [9]. In mammals, HF has displayed therapeutic promise for conditions such as muscular dystrophy [10], hepatic fibrosis [11], and cancer, by inhibiting tumor metastasis [12,13]. HF has also been shown to protect mice from ischemia-reperfusion–induced inflammation [2]. Recent studies have demonstrated that HF acts by inhibiting the prolyl-tRNA synthetase activity of glutamyl-prolyl-tRNA synthetase (EPRS) [14]. HF actively competes with proline for the active site of prolyl-tRNA synthetase, resulting in the accumulation of uncharged tRNA^pro^. This mimicks conditions of cellular proline deficiency, which in turn triggers simultaneous activation of the GCN2–AAR pathway [14].

Recently, system-wide analysis of immunological responses to yellow fever vaccine (YF-17D) identified gene signatures encompassing GCN2 [15], which plays a critical role in programming YF-17D–induced T-cell responses [16]. Furthermore, recent studies suggest that HF-mediated activation of the GCN2–AAR pathway inhibits T helper 17 cell (Th17) responses and protects mice from Th17-associated pathologies in a mouse model of experimental autoimmune encephalomyelitis (EAE) [17]. Also, GCN2 has recently been shown to play a central role in controlling intestinal inflammation through the inhibition of interleukin 1β (IL-1β), a key proinflammatory mediator [4]. While the connections between the GCN2 and AAR pathways controlling immune responses is evident, the mechanism underlying its association with regulation of IL-1β and other inflammatory mediators remains poorly understood. The production of IL-1β includes the processing of inactive IL-1β precursor protein (31 kDa) into the bioactive or mature IL-1β (17 kDa) and release through K^+^ efflux-dependent mechanisms, which can be triggered by ATP, a known inducer of potassium efflux [18]. Processing of active IL-1β is largely dependent on caspase-1 activation triggered by inflammasome activation following toll-like receptor (TLR) stimulation. Here, we show that HF dampens endotoxin-induced IL-1β production in macrophages by a novel mechanism of PTR and translation reprogramming. HF-elicited PTR events resulted in the repositioning of IL-1β mRNA transcripts from polysomes to riboclusters, such as SGs, in a GCN2-dependent manner. Also, IL-1β mRNAs targeted to SGs are further cleared through the induction of autophagy. HF impaired the processing and secretion of mature IL-1β in lipopolysaccharide (LPS)-primed macrophages by controlling the reactive oxygen species (ROS) production and inflammasome activation. Furthermore, HF protected mice from dextran sulfate sodium (DSS)–induced intestinal inflammation. Collectively, these findings highlight a crucial role for the GCN2–AAR pathway in the regulation of IL-1β production and unveil a novel mechanism through which AAR modulates inflammatory responses.

## Results

### HF ameliorates LPS-induced IL-1β production in macrophages by affecting mRNA stability and processing of mature IL-1β

**Fig 1:**
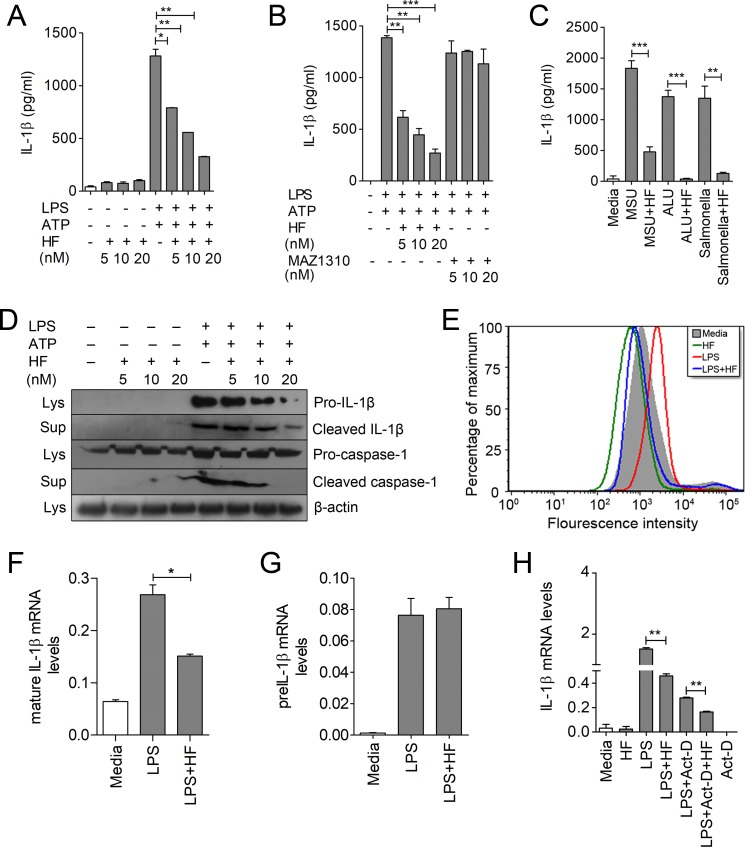
HF ameliorates LPS-induced production of IL-1β in macrophages by affecting mRNA stability and processing of mature IL-1β. (A–B) IL-1β production from LPS (500 ng/ml)-primed or -unprimed BMDMs treated with different concentrations of HF or MAZ1310 (control) for 6 h. ATP (5 mM) was added to the LPS-stimulated macrophage cultures for 30 min at the end of time point (S1 Data). Statistical significance was determined by student *t* test. *P ≤ 0.05, **P ≤ 0.005, ***P ≤ 0.0005. (C) IL-1β production from LPS-primed macrophages stimulated with MSU (150 ug/ml), or ALU (200 ug/ml) for 6 h, or infected with *S*. *typhimurium* (MOI 10) in presence or absence of HF (S1 Data). (D) IL-1β and caspase-1 (pro and active) expression by immunoblot analysis from LPS-primed BMDMs treated with HF as indicated; β-actin was used as loading control. (E) ROS levels detected by CM-H2DCFDA staining in macrophages treated with HF or LPS plus HF. (F, G) qRT-PCR analysis of mature IL-1β and pre–IL-1β mRNA levels in J774A.1 cells stimulated with LPS or LPS plus HF (S1 Data). (H) Analysis of IL-1β mRNA levels by qRT-PCR in LPS-primed macrophages treated with Act-D for 2 h followed by HF treatment for an additional 2 h (S1 Data). *P ≤ 0.05, **P ≤ 0.005, ***P ≤ 0.0005 were considered statistically significant. Data are representative of 1 of 3–4 independent experiments. Act-D, actinomycin-D; ALU, aluminum hydroxide; BMDM, bone marrow–derived macrophage; HF, Halofuginone; IL-1β, interleukin 1β; LPS, lipopolysaccharide; Lys., cell lysates; MOI, multiplicity of infection; MSU, monosodium urate; qRT-PCR, quantitative reverse transcription PCR; ROS, reactive oxygen species; Sup., culture supernatant.

Recent studies have shown that HF exerts its therapeutic benefits through inhibition of Th17 [17]; however, its role in controlling innate inflammatory responses is not yet clear. To investigate the immunomodulatory function of HF, LPS-primed bone marrow–derived macrophages (BMDMs) were treated with nontoxic concentrations of HF for different time points (S1 Fig). We observed a substantial reduction of IL-1β in a dose- and time-dependent manner by HF (Fig 1A and S2A and S2B Fig), while tumor necrosis factor α (TNF-α) showed a decrease, and there was no suppressive effect on IL-6 during HF treatment (S2B and S2C Fig). HF treatment inhibited IL-1β production in peritoneal as well as transformed macrophages (S2D and S2E Fig). By contrast, treatment of LPS-primed macrophages with MAZ1310, an inactive derivative of HF, did not show any effect on IL-1β production, indicating that the observed effects are specific to HF (Fig 1B). Next, we assessed the effect of HF on damage associated molecular patterns (DAMPs)-induced IL-1β production. HF substantially impaired IL-1β production in macrophages treated with inflammasome activators such as aluminum hydroxide (ALU) or monosodium urate (MSU) (Fig 1C). Apart from its effects on model TLR ligand (which represent less complex stimuli than bacteria or viruses)–induced IL-1β, HF also inhibited IL-1β production by BMDMs infected with *Salmonella typhimurium* (Fig 1C). Next, we examined the effect of HF on IL-1β processing and secretion. Results in (Fig 1D) show that HF substantially reduces mature IL-1β induced by LPS plus ATP, as well as cleaved caspase-1, in the supernatant of treated cells. These results indicate that HF affects IL-1β production by interfering with inflammasome activation.

Inflammasomes are multiprotein complexes activated during microbial infection or stress, and they elicit caspase-1 activation and the processing of IL-1β [19]. Although the mechanisms underlying inflammasome activation are still unclear, one of the proposed models suggests that cellular ROS play a pivotal role in the inflammasome activation [20]. Although the source of ROS is still unclear, studies suggest an association with NADPH oxidase activation [21]. We therefore examined the effect of HF on ROS production by stimulating BMDMs with LPS for 3h, followed by HF treatment. We found a significant reduction of ROS levels in LPS plus HF–stimulated macrophages compared to LPS alone (Fig 1E), suggesting that HF may limit IL-1β secretion through the suppression of the ROS generation as well.

TLR family members TLR2 and TLR4 recognize bacterial components and play a crucial role in the antibacterial response [22], and our results suggest that inhibition of IL-1β by HF is pronounced when triggered by a TLR4 ligand compared to TLR2 ligands (S2F Fig). Concomitantly, HF did not show much effect on IL-1β in the macrophages primed with viral nucleotide-sensing TLR ligands TLR3, TLR9, and TLR7. HF dramatically decreased pro–IL-1β levels in the cell lysates, whereas the pro–caspase-1 levels were not affected by HF in LPS- and LPS plus HF–stimulated macrophages (Fig 1D, S2G Fig), indicating that HF affects IL-1β not only at the level of processing but also at the level of expression of pro–IL-1β. To examine the mechanism by which pro–IL-1β is reduced by HF, we examined the effect of HF on mRNA for IL-1β and other cytokines by quantitative reverse transcription PCR (qRT-PCR). The results revealed a significant decrease in the levels of mature IL-1β and IL-18 mRNA compared to other cytokines (Fig 1F, S3A Fig); however, pre–IL-1β mRNA was not affected (Fig 1G). These data suggest that the effect of HF on IL-1β mRNA might not be at the transcriptional level. We next speculated that HF might target IL-1β partly through modulation of transcriptional/PTR events. To investigate whether HF-induced inhibition of IL-1β mRNA is a result of PTR, BMDMs were treated with LPS for 2 h, followed by treatment with the transcriptional inhibitor actinomycin-D (Act-D) for 2h before the addition of HF. Surprisingly, we observed a remarkable reduction in IL-1β mRNA transcripts in LPS plus HF–stimulated macrophages compared to LPS alone in Act-D–primed cells (Fig 1H). HF also inhibited IL-1β mRNA when IL-1β was constitutively expressed in human embryonic kidney cells 293T (HEK293T) by transfection of an IL-1β–expressing plasmid driven by the cytomegalovirus (CMV) promoter independent of TLR signaling (S3B Fig). Translational control of IL-1β by HF was further confirmed by inhibiting translation in BMDMs with cycloheximide, a translational inhibitor, prior to LPS and HF treatment (S3C Fig). Altogether, these results suggest that HF controls IL-1β production by affecting inflammasome activation and triggering of PTR events without significant effect on transcriptional reprogramming.

### HF attenuates LPS-induced IL-1β production through GCN2-dependent activation of riboclustering, such as SG formation

**Fig 2:**
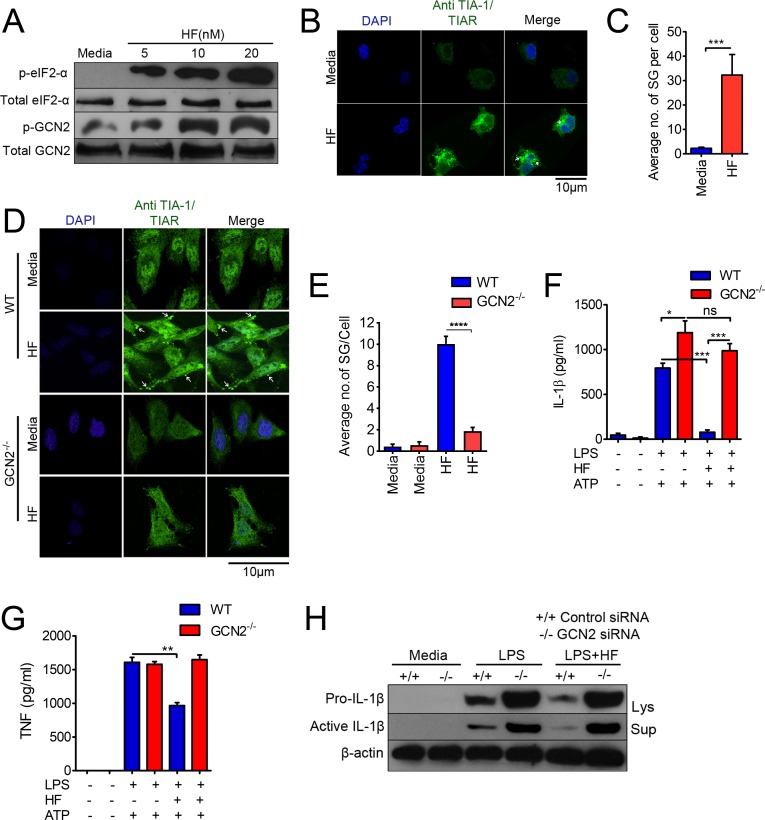
HF attenuates LPS-induced IL-1β production through GCN2-dependent activation of PTR events such as riboclustering and SG formation. (A) Immunoblot analysis of GCN2 and eIF2-α phosphorylation in the lysates of macrophages treated or untreated with varying concentrations of HF for 3 h. (B) Confocal microscopy imaging of SGs, indicated by white arrows in macrophages stimulated with HF (20 nM) for 3 h; nuclei were stained with DAPI (blue). Scale bars, 10 μm. (C) Quantification of average number of SGs per cell, from 5 different fields taken from the results in panel B (S1 Data). (D) Confocal microscopy imaging of SGs in WT (top) or GCN2^−/−^ MEFs (bottom). (E) Quantification of an average number of SGs per cell in WT or GCN2^−/−^ cells treated with HF (S1 Data). (F, G) IL-1β (panel F) or TNF-α (panel G) levels by ELISA in the culture supernatants of WT or GCN2^−/−^ BMDMs primed with LPS for 3 h followed by HF treatment. ATP (5 mM) was added to the cultures for 30 min at the end of the experiment (S1 Data). *P < 0.05, **P < 0.005. (H) J774A.1 macrophages were transfected with either control siRNA or GCN2 siRNA and stimulated with LPS (500 ng/ml) or LPS plus HF (20 nM); ATP (5 mM) was added to the cultures for 30 min at the end of the experiment. IL-1β (pro and active) forms were examined in the cell lysates and culture supernatants by immunoblotting. Data are representative of 1 of 3 similar experiments. BMDM, bone marrow–derived macrophage; eIF2, eukaryotic initiation factor 2; GCN2, general control nonderepressible 2 kinase; HF, Halofuginone; IL-1β, interleukin 1β; LPS, lipopolysaccharide; Lys, cell lysates; MEF, mouse embryonic fibroblast cell; PTR, post-transcriptional reprogramming; SG, stress granule; siRNA, small interfering RNA; Sup, culture supernatant; TIA-1, T cell–restricted intracellular antigen-1; TIAR, TIA-1–related; TNF, tumor necrosis factor; WT, wild-type.

Post-transcriptional control of cytokines and other immune effector mRNAs ensures rapid temporal or spatial changes in protein expression in response to changing environmental cues [23], mediated by sensors such as GCN2, PERK, PKR, and HRI, which sense particular stress conditions [5]. GCN2 senses amino acid deprivation and triggers activation of the ISR pathway, which in turn programs translatability and/or decay of mRNAs as per cellular requirements through riboclustering [7]. Recent studies have shown that HF-induced biological activities are attributed to the activation of the AAR pathway [14]. Therefore, we next studied the role of the GCN2–AAR pathways in the ability of HF to regulate IL-1β. We observed elevated phosphorylation of GCN2 and eIF2-α in macrophages with increasing concentrations of HF (Fig 2A), which is in agreement with earlier studies performed on T cells [17], fibroblasts, and epithelial cells [24]. Concomitantly, the formation of punctate SGs increased with HF treatment (Fig 2B and 2C and S4 Fig). In addition, GCN2^−/−^mouse embryonic fibroblast cells (MEFs) showed impaired ability to form SGs during HF treatment compared to wild-type (WT) cells (Fig 2D and 2E), suggesting that HF-induced SG formation is GCN2 dependent. To elucidate the role of GCN2 in HF-induced negative regulation of IL-1β production, BMDMs isolated from WT or GCN2^−/−^ mice were primed with LPS followed by HF treatment. While LPS plus ATP treatment enhanced IL-1β production in GCN2^−/−^ BMDMs when compared to WT cells, HF only suppressed IL-1β production in WT but not in GCN2^−/−^macrophages (Fig 2F). On the otherhand, HF modestly decreased LPS-induced TNF-α in WT cells, and HF did not affect LPS-induced TNF-α levels in GCN2^−/−^ cells (Fig 2G). Similar results were observed in levels of pro and active IL-1β protein as well as mature IL-1β and TNF-α mRNA in macrophages with transiently silenced GCN2 (Fig 2H, S5A, S5B and S5C Fig).The observed effects of HF were specific to GCN2 because PKR-specific small interfering RNA (siRNA)-mediated knockdown of PKR—another eIF2 kinase—did not affect HF’s ability to reduce IL-1β production, indicating a PKR-independent effect (S6 Fig). HF induces serine 51 (Ser51) phosphorylation of eIF2-α via GCN2, and so we examined the effect of eIF2-α on IL-1β by silencing eIF2-α in macrophages. We found that HF significantly suppressed IL-1β production only in control cells, while no significant inhibition was observed in eIF2-silenced macrophages (S5D Fig). Altogether, these results suggest that HF limits IL-1β production by activating the GCN2–eIF2-α axis.

### HF promotes IL-1β mRNA degradation by targeting them to SGs through recruitment of TIA-1/TIAR

**Fig 3:**
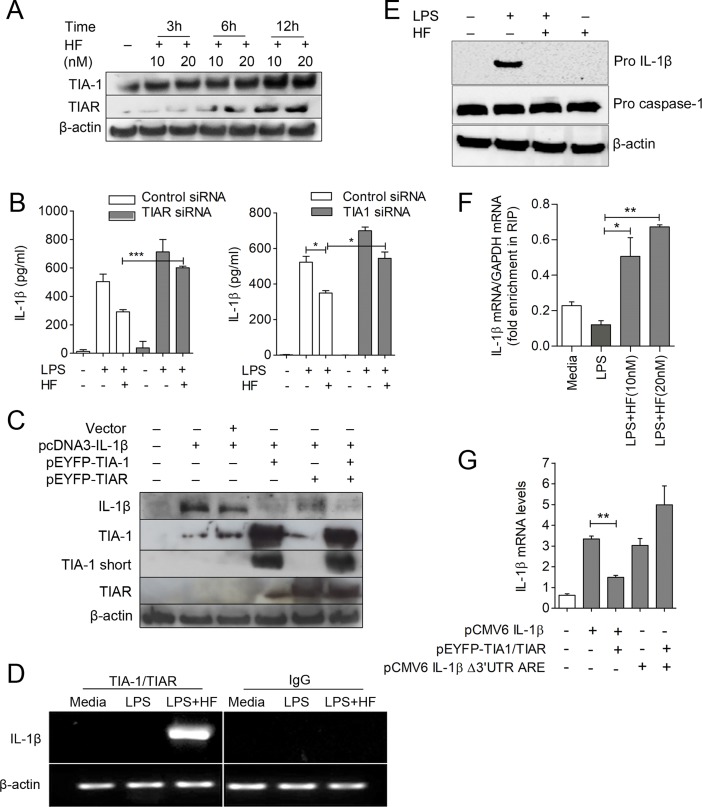
HF treatment promotes IL-1β mRNA degradation by targeting it to SGs through recruitment of TIA-1/TIAR. (A) Immunoblot analysis of RBPs, TIA-1/TIAR, in the lysates of J774A.1 macrophages, left untreated or treated with varying concentrations of HF for 3-h, 6-h, and 12-h time points. β-actin was used as loading control. (B) J774A.1 macrophages were transfected with either control siRNA or TIA-1/TIAR siRNA. After 24 h, cells were either left untreated or stimulated with LPS (500 ng/ml) or LPS (500 ng/ml) plus HF (20 nM) for 6 h, ATP (5 mM) was added to the macrophage cultures for 30 min at the end of time point and were assayed for IL-1β levels in culture supernatants by ELISA (S1 Data). (C) IL-1β expression in the whole-cell lysates of HEK293T cells transiently expressing various combinations of plasmids (pcDNA3, pcDNA3-IL-1β, and pEYFP-TIA-1/TIAR) as indicated (top). β-actin was used as loading control. (D) RT-PCR analysis of IL-1β expression in RIP material (pull-down using TIA-1/TIAR or IgG) of lysates from J774A.1 macrophages left untreated or treated with LPS or LPS plus HF. (E) Immunoblot analysis of pro–IL-1β or pro–caspase-1 expression in macrophages treated with LPS or LPS plus HF; β–actin was used as a loading control. (F) qRT-PCR analysis of IL-1β mRNA in the RIP material from LPS-primed J774A.1 macrophages treated with the indicated concentrations of HF (S1 Data). (G) qRT-PCR analysis of IL-1β mRNA in HEK293T cells transfected with constructs pCMV6-IL1β or pCMV6 IL-1β Δ3'UTR ARE, or cotransfected along with pEYFP-TIA-1/TIAR (S1 Data). Statistical significance was determined by student *t* test. *P ≤ 0.05, **P ≤ 0.005, ***P ≤ 0.0005. Data are representative of 1 of 3 independent experiments. HEK293T, human embryonic kidney cells 293T; HF, Halofuginone; IgG, immunoglobulin G; IL-1β, interleukin 1β; LPS, lipopolysaccharide; qRT-PCR, quantitative reverse transcription PCR; RBP, RNA-binding protein; RIP, RNA immunoprecipitation; siRNA, small interfering RNA; TIA-1, T cell–restricted intracellular antigen-1; TIAR, TIA-1–related.

Having observed that HF induces SG formation, we next studied the mechanisms involved by monitoring the expression pattern of the SG proteins TIA-1/TIAR. TIA-1/TIAR are RBPs involved in the regulation of mRNA transcripts. HF alone induces expression of TIA-1/TIAR in a time- and concentration-dependent manner (Fig 3A). A similar increase was observed in LPS plus HF–or HF only–stimulated macrophages, while LPS alone shows no effect on TIA-1/TIAR levels (S5E Fig). To elucidate whether HF-induced TIA-1/TIAR plays a role in the suppression of IL-1β expression, we silenced the expression of TIA-1 or TIAR in macrophages using smart pool TIA-1 or TIAR siRNAs. These cells were then primed with LPS followed by treatment with HF. We observed an enhancement of IL-1β production both at the protein and mRNA levels in TIA-1/TIAR–silenced macrophages in response to LPS stimulation, and HF-induced suppression of IL-1β production is reduced in these cells (Fig 3B and S5F and S5G Fig). Conversely, we overexpressed TIA-1/TIAR along with the IL-1β in HEK293T cells, driven by heterologous promoters, and examined IL-1β protein expression by immunoblotting. We detected reduced IL-1β expression in the lysates of HEK293T cells overexpressing TIA-1/TIAR compared to vector controls (Fig 3C). These data strongly suggest that TIA-1/TIAR play a significant role in the regulation of IL-1β expression. Furthermore, we studied whether eIF2-α signaling plays any significant role in controlling the expression levels of TIA-1/TIAR. Our results suggest eIF2-α–dependent expression of TIA-1/TIAR (S5H Fig). IL-1β mRNA bear ARE sequences in the 3’UTR [7], which tempted us to speculate that IL-1β mRNA might recruit RBPs—including TIA-1/TIAR—through these ARE sequences and lead to SG formation during GCN2-eIF2 activation. To test this hypothesis, we stimulated macrophages with LPS or LPS plus HF and prepared cell lysates for RNA immunoprecipitation (RIP) using TIA-1/TIAR antibodies or immunoglobulin G (IgG) control. Using both by qRT-PCR and RT-PCR analysis, we found that TIA-1/TIAR pull-downs were associated with IL-1β and TNF-α—but not caspase-1 and IL-6 transcripts—in the LPS plus HF–treated groups but not the LPS-alone groups (Fig 3D and S7 Fig). Control IgG failed to pull down IL-1β transcripts in both LPS- and LPS plus HF–stimulated macrophages. Furthermore, nontarget β-actin was amplified to the same extent in both groups (Fig 3D), indicating the presence of equal amounts of nonspecific contaminating mRNAs in both the immunoprecipitation (IP) material as observed in the earlier studies [25]. Because we noticed that HF induced a significant increase in IL-1β transcripts in the RIP material, we examined its impact on pro–IL-1β expression in the cell lysates. Our results show a substantial decrease in pro–IL-1β expression in LPS plus HF–treated compared to LPS alone–treated cells, whereas pro–caspase-1 levels were comparable in both LPS- and LPS plus HF–treated groups (Fig 3E). Furthermore, when RIP was performed using TIA-1/TIAR antibodies in LPS-primed macrophages treated with different concentrations of HF, we observed a proportionate increase in IL-1β transcripts coprecipitating in a HF-dose–dependent manner (Fig 3F). Because TIA-1/TIAR bind to ARE sequences found at the 3’UTRs of mRNAs and regulate their expression, we reasoned that IL-1β mRNA carrying a mutation in 3’UTR ARE elements might not be affected by TIA-1/TIAR. To test this speculation, we overexpressed IL-1β mRNAs carrying WT (no mutation in AREs of 3’UTR) or ARE mutation (Δ3’UTR) in HEK293T cells using a heterologous promoter (CMV), along with TIA-1/TIAR, and assessed the expression of IL-1β mRNA. We found that TIA-1/TIAR overexpression reduced IL-1β mRNA (as detected by qRT-PCR) only in cells expressing WT plasmid but not in the cells expressing the ARE-deleted plasmid (Fig 3G). These results clearly demonstrate that HF triggers riboclustering through recruitment of TIA-1/TIAR proteins to the ARE sequences in the IL-1β mRNA and facilitates its movement to cytoplasmic caches of SGs for degradation.

### IL-1β targeted to SGs are degraded by autophagy during HF treatment

**Fig 4:**
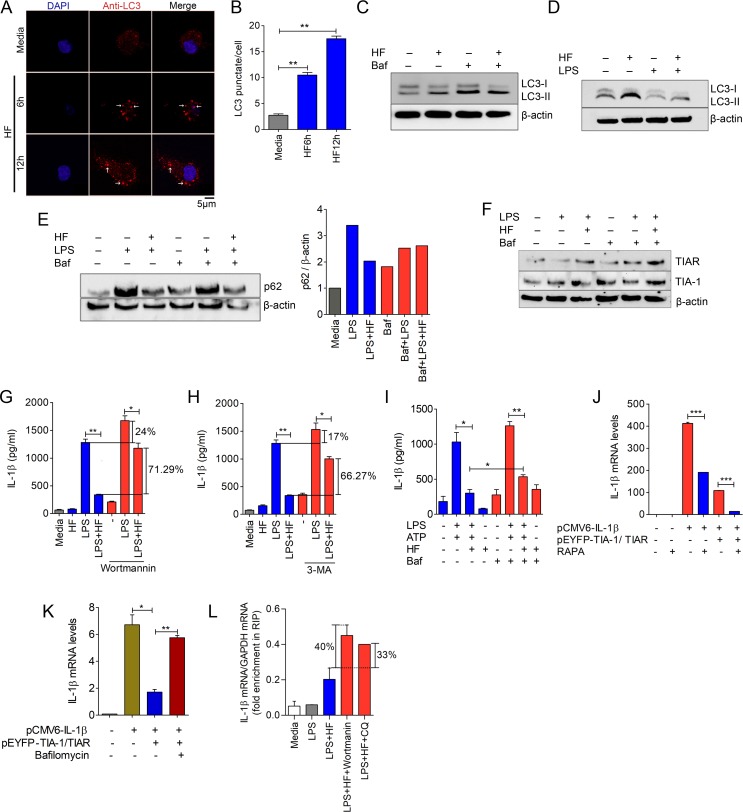
IL-1β transcripts targeted to SGs are degraded by the activation of autophagy during HF treatment. (A) Confocal microscopy imaging of LC3 punctates (indicated by white arrows) in BMDMs left untreated (media control) or treated with HF (20 nM) at time points indicated. Scale bars, 5 μm. (B) LC3 punctate counts per cell (S1 Data). (C) Immunoblot analysis of autophagy marker, LC3 in J774A.1 macrophages left untreated or treated with HF in the presence or absence of autophagy inhibitor Baf. (D) Immunoblotting of LC3 in the lysates of J774A.1 macrophages treated with LPS plus HF or LPS alone. ATP was added to the cultures for 30’ at the end of the experiment. β-actin was used as a loading control. (E) Immunoblot analysis of p62/SQSTM1 expression in J774A.1 macrophages stimulated with LPS plus ATP or LPS plus HF plus ATP in the presence or absence of autophagy inhibitor Baf (S1 data). (F) Immunoblot analysis of TIA-1/TIAR expression in J774A.1 macrophages treated with HF or LPS in the presence or absence of Baf (10 nM). (G–I) Quantification of IL-1β levels using ELISA in culture supernatants of macrophages stimulated with LPS (500 ng/ml) alone or LPS (500 ng/ml) plus HF (20 nM) in the presence or absence of pharmacological inhibitors of autophagy, 3-MA, Wortmannin (500 nM), or Baf (10 nM). ATP (5 mM) was added for 30 min at the end of the experiment (S1 Data). Statistical significance was determined by student *t* test. *P ≤ 0.05, **P ≤ 0.005. (J, K) qRT-PCR analysis of IL-1β in HEK293T cells transfected with pCMV6-IL-1β or cotransfected with pCMV6-IL-1β plus pEYFP-TIA-1/pEYFP-TIAR followed by rapamycin (100 nM) or Baf treatment (S1 Data). ***P ≤ 0.0005. (L) qRT-PCR analysis of IL-1β mRNA in the RIP material of LPS-primed macrophages treated with HF in presence of Wortmannin (500 nM) or CQ (25 μM) (S1 Data). Data are representative of 1 of 3 independent experiments. 3-MA, 3-methyl adenine; Baf, Bafilomycin A1; BMDM, bone marrow–derived macrophage; CQ, chloroquine; HEK293T, human embryonic kidney cells 293T; HF, Halofuginone; IL-1β, interleukin 1β; LPS, lipopolysaccharide; p62/SQSTM1, sequestosome 1; qRT-PCR, quantitative reverse transcription PCR; RIP, RNA immunoprecipitation; SG, stress granule; TIA-1, T cell–restricted intracellular antigen-1; TIAR, TIA-1–related.

Recent studies have shown that mice deficient in autophagy proteins, autophagy-related 16-like 1 (Atg16L1) or autophagy related protein 7 (Atg7) are prone to enhanced IL-1β production [26]. It has also been demonstrated that SGs are cleared by autophagy [27]. Therefore, we examined the status of autophagy during HF stimulation by monitoring autophagy marker, microtubule-associated protein 1A/1B light chain 3 (LC3). We found that autophagy vesicles (revealed by LC3 punctate staining) were enhanced at 6 and 12 h of HF stimulation (Fig 4A and 4B). Furthermore, analysis of conversion of LC3-I to a membrane-bound form, LC3-II [28], in the cell lysates by immunoblotting revealed an increase in the conversion and accumulation of LC3-II in response to HF (Fig 4C, and S8A Fig). Furthermore, LC3-II conversion was enhanced in LPS plus HF–treated cells compared to LPS alone–treated cells (Fig 4D).

Next, we assessed the autophagy flux by monitoring the levels of LC3-II and sequestosome 1 (p62/SQSTM1; a well-known autophagy substrate) in the presence or absence of autophagy inhibitors chloroquine (CQ) or Bafilomycin A1. LPS is known to induce expression of p62 [29], and we found that there was a dramatic reduction of p62 in macrophages treated with LPS plus HF plus ATP compared to cells treated with LPS plus ATP alone (Fig 4E). This effect of HF on p62 levels was inhibited by Bafilomycin A1 in LPS plus HF–treated cells (Fig 4E). Accumulating evidence suggests that p62 and LC3-II are degraded by autophagy during long-term starvation, i.e., from 2 h [30,31]. CQ or Bafilomycin A1 strongly inhibited basal autophagy, marked by the accumulation of LC3 and p62, and HF treatment caused a decrease in p62 and LC3-II levels in the presence of CQ (possibly due to activation of autophago-lysosomal degradation), but not in Bafilomycin A1–treated cells (Fig 4C and S8A and S8B Fig), suggesting that Bafilomycin A1 exhibits more lysosomal inhibitory effect than CQ, as previously described [31]. However, levels of LC3-II are still higher during HF treatment in the presence of CQ compared to cells treated with HF in the absence of CQ (S8A Fig). Together, these data clearly indicate that HF triggers autophagy flux. Furthermore, similar to amino acid starvation–induced p62 mRNA expression [32], HF caused up-regulation of p62 expression, which is dependent on GCN2 (S8C and S8D Fig). Collectively, these results suggest that HF triggers induction of autophagic processes. We next examined whether autophagy has any effect on expression of SG proteins TIA-1/TIAR in macrophages treated with LPS plus HF. Our results show that LPS plus HF in presence of Bafilomycin A1 induces the accumulation of TIA-1/TIAR (Fig 4F). Furthermore, TIA-1/TIAR colocalizes with autophagy marker LC3 as revealed by immunofluorescence microscopy (S9A Fig). Together, these results indicate that autophagy mediates the clearance of SGs.

Next, we investigated whether manipulation of autophagy had any effect on HF-induced suppression of IL-1β expression in LPS-primed macrophages. Pharmacological inhibition of autophagy by using phosphotidyl inositol 3 kinase inhibitors 3-methyl adenine (3-MA) or Wortmannin [33,34], lysosomal inhibitor Bafilomycin A1, or knockdown of autophagy gene ATG16L1 in macrophages resulted in elevated levels of IL-1β in LPS plus HF–stimulated cells compared to their controls (Fig 4G–4I and S9B Fig). These results suggest that HF-induced autophagy plays a significant role in the suppression of IL-1β. To explore the mechanism, IL-1β was overexpressed using a heterologous promoter in HEK293T cells alone, or along with plasmids expressing TIA-1 and TIAR. Following transfection, cells were treated with autophagy inhibitor (Bafilomycin A1) or activator (rapamycin) and levels of IL-1β mRNA were monitored. We observed a significant reduction in IL-1β expression in TIA-1/TIAR–overexpressing cells—which was further decreased by rapamycin treatment—whereas Bafilomycin A1 reversed the inhibition (Fig 4J and 4K). RIP also revealed an increase in IL-1β mRNA association with TIA-1/TIAR under the conditions of inhibition of autophagy (Fig 4L). Recent studies have shown that activation of autophagy decreases mitochondria-derived oxidative stress (a potent inflammasome activator) by clearing damaged mitochondria, thereby affecting IL-1β levels [29]. We therefore measured the levels of mitochondrial ROS using MitoSOX in LPS-primed macrophages during HF or control MAZ1310 treatment. HF significantly inhibited MitoSOX production (S9C and S9D Fig). Together, these data suggest that HF-induced autophagy triggers IL-1β suppression via degradation of IL-1β mRNA transcripts bound to SG components, TIA-1/TIAR, and by reducing inflammasome activation signals.

### HF-mediated activation of autophagy is dependent on GCN2

**Fig 5:**
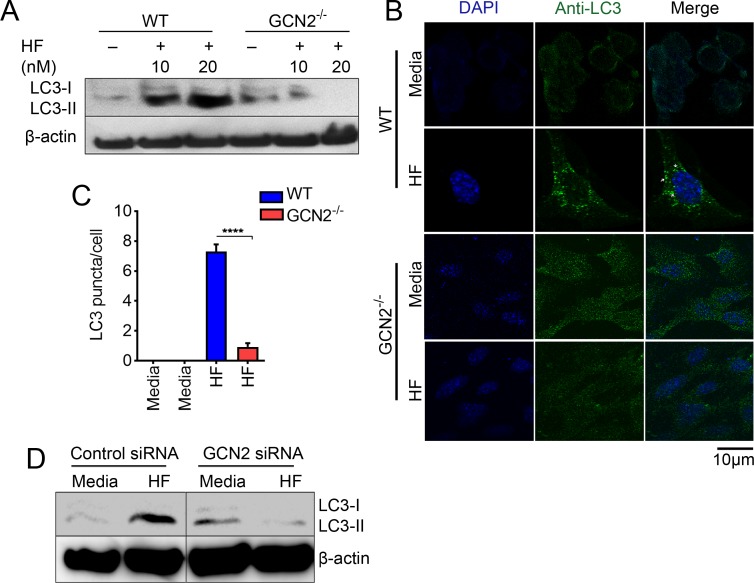
HF-mediated activation of autophagy is dependent on GCN2. (A) Immunoblot analysis of LC3 protein (LC3II conversion, membrane-bound form) in lysates of WT or GCN2^−/−^MEFs left untreated or treated with HF at concentrations specified for 6 h. β-actin was used as loading control. (B) Confocal microscopy imaging of LC3 protein (punctates in green) in WT (top) or GCN2^−/−^ MEFs (bottom) left untreated or treated with HF (20 nM) for 6 h; nuclei were stained with DAPI (blue). Scale bars, 10 μm. (C) LC3 punctate per cell count in WT or GCN2^−/−^ MEFs (S1 Data). ****P ≤ 0.0001. (D) Immunoblot analysis of LC3 in control or GCN2 siRNA-transfected macrophages treated with HF (20 nM). Data are representative of 1 of 3–4 independent experiments. GCN2, general control nonderepressible 2 kinase; HF, Halofuginone; LC3, microtubule-associated protein 1A/1B light chain 3; MEF, mouse embryonic fibroblast cell; siRNA, small interfering RNA; WT, wild-type.

Recent studies have demonstrated a link between AAR and autophagy in yeast and mammals [35]. The above results support the previous findings [17], showing that AAR mimetics such as HF act through the activation of GCN2. However, the link between HF-induced autophagy and GCN2 remains unclear. Therefore, we examined the conversion of LC3 in GCN2^−/−^ MEFs treated with varying concentrations of HF as well as in lysates of GCN2-silenced macrophages during HF treatment. We observed a dose-dependent increase in LC3-I to LC3-II conversion in the WT MEFs treated with HF, but not in GCN2^−/−^MEFs (Fig 5A). Furthermore, analysis of autophagosomal puncta (LC3II accumulation) in the cytosol via confocal microscopy revealed that the appearance of LC3^+^ dots was enhanced in WT but not in GCN2^−/−^ MEFs exposed to HF (Fig 5B and 5C). Similar results in LC3 conversion were observed in GCN2 siRNA–transfected cells compared to its control siRNA–transfected macrophages (Fig 5D). Conversely, when GCN2 was overexpressed in HEK293T cells and the transfected cells starved for different time periods, LC3 conversion was significantly increased compared to cells cultured in normal media (S8E Fig). These results indicate that HF-triggered induction of autophagy is dependent on GCN2.

### HF mitigates the severity of DSS-induced colitis in mice

**Fig 6:**
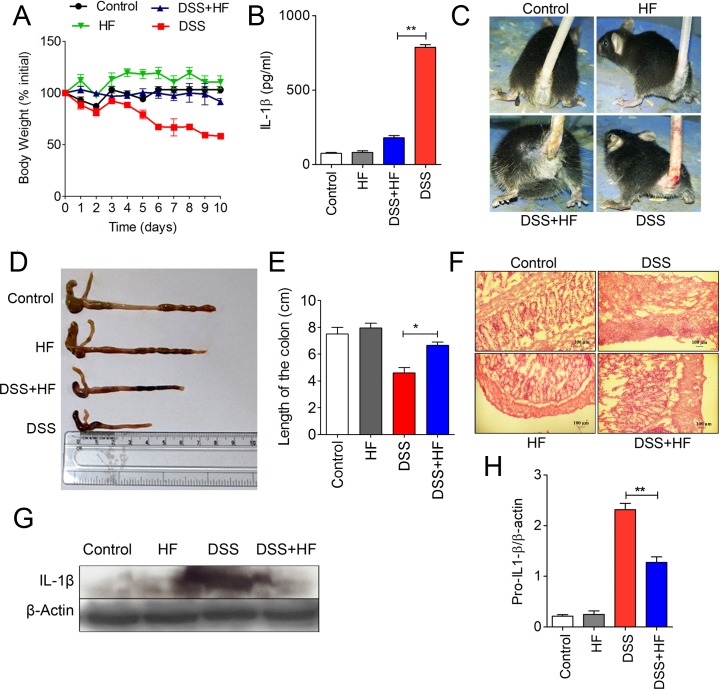
HF mitigates the severity of DSS-induced colitis in mice. (A) Body weight (percentage of initial body weight) of mice (*n* = 5) (S1 Data). (B) Quantification of IL-1β levels by ELISA in serum samples of the indicated mice (S1 Data). **P ≤ 0.0015. (C) Visualization of rectal bleeding. (D) Visualization of typical colon length in control, HF-, DSS-, and DSS plus HF–treated mice. (E) Measurement of colon length (cm) (S1 Data). (F) Visualization of mucosal epithelium erosion and crypt loss in colon sections (HE-stained) as indicated. (G) Immunoblot analysis of IL-1β levels in the large intestine tissue samples. (H) Densitometric analysis of pro–IL-1β levels from colon tissues of mice subjected to treatments as indicated (*n* = 4) (S1 Data). β-actin was used as loading control. Data are representative of 1 of 3 separate experiments. DSS, dextran sulfate sodium; HE, hematoxylin–eosin; HF, Halofuginone; IL-1β, interleukin 1β.

Anomalous expression of proinflammatory cytokines, particularly IL-1β, has been shown to be associated with colitis [36,37]. Furthermore, transcriptome meta-analysis performed on publicly available microarray datasets (Gene Expression Omnibus [GEO], Array Express) from samples of human inflammatory bowel disease (IBD) patients reveals that GCN2 was significantly down-regulated in the peripheral blood mononuclear cells (PBMCs) of both ulcerative colitis (UC) and Crohn’s disease (CD), whereas it was up-regulated in colon tissues (S10A–S10C Fig). Given our findings that HF inhibits IL-1β, we therefore investigated the therapeutic potential of HF in a murine model of DSS-induced colitis. We induced colitis in C57BL/6J mice by oral administration of 5% DSS in drinking water and found that daily intraperitoneal injections of HF (0.2 mg/kg) prevented the loss of body weight observed in the absence of HF (Fig 6A). Levels of IL-1β in the serum and the colonic tissues of DSS plus HF–treated mice were markedly reduced compared to the DSS-treated mice (Fig 6B, 6G and 6H). Furthermore, we observed that HF treatment ameliorated DSS-induced rectal bleeding (Fig 6C). Shortening of colon length, an important characteristic of colitis, was also reduced in DSS plus HF–administered mice compared to only DSS-treated mice (Fig 6D and 6E). Microscopic examination of colon tissue sections from DSS plus HF–treated mice showed that HF prevented the DSS-induced inflammation, crypt loss, and epithelial damage (Fig 6F). These results suggest the therapeutic potential of HF-mediated GCN2–AAR pathways in controlling intestinal inflammation.

## Discussion

Amino acid restriction is associated with enormous health benefits, including longevity of lifespan [38], acute stress resistance [39], increased insulin sensitivity, and modulation of inflammation [2,3] in yeast to nonhuman primates. However, the clinical benefits of amino acid restriction to humans has yet to be achieved. Our study has established that pharmacological activation of AAR with a plant-derived small biomolecule HF modulates innate inflammatory responses by activating the homeostatic ISR pathway. Our findings demonstrate that HF-induced cytoprotective AAR is dependent on GCN2 because HF failed to trigger AAR in GCN2-ablated cells. The HF-induced AAR pathway triggers a significant reduction in LPS-induced IL-1β, with little inhibitory effect on another inflammatory cytokine TNF-α and no effect on IL-6. Although HF has been previously shown to activate GCN2 kinase, the molecular basis by which it controls IL-1β and other inflammatory mediators has been poorly understood. Our study has revealed the mechanisms by which HF suppresses IL-1β expression in response to LPS.

IL-1β is a potent inflammatory cytokine with diverse cellular and physiological functions [40] and as such, requires stringent regulatory mechanisms to control its expression and secretion. IL-1β is translated as an inactive 31 kD precursor protein (pro–IL-1β) in response to TLR4 stimulation, which is further cleaved into mature bioactive IL-1β (p17) by caspase-1, triggered by inflammasome activation [41]. Our study shows that HF impairs processing of mature IL-1β by reducing inflammasome activation, which could be partly attributed to reduced ROS production. We further show that HF reduces mature IL-1β transcripts, but not its transcription. These findings suggest that HF inhibits IL-1β production by programming PTR/translational events. It is notable that dexamethasone was recently reported to inhibit IL-1β production by programming PTR events, without much effect on transcriptional machinery function, similar to what we find for HF [42]. Various studies have reported that IL-1β plays a crucial role in the commitment of Th17 cells [4], and it was recently demonstrated that HF inhibits the Th17 response and protects mice from EAE-associated inflammation through the activation of the GCN2–AAR pathway in T cells [17]. However, how HF controls innate regulation of Th17 cell responses remained elusive. Our findings suggest that HF likely determines the effector commitment of Th17 cells in part by also inhibiting IL-1β production through programming PTR/translational events. In line with the above studies, it was recently shown that the AAR sensor GCN2 plays a pivotal role in controlling intestinal inflammation [4]. HF preconditioning protects the mice from surgical stress–induced inflammation in a mouse model of ischemia-reperfusion injury [2]. Our study mechanistically supports previously published work highlighting the beneficial aspects of HF preconditioning or AA restriction in the context of inflammation.

**Fig 7:**
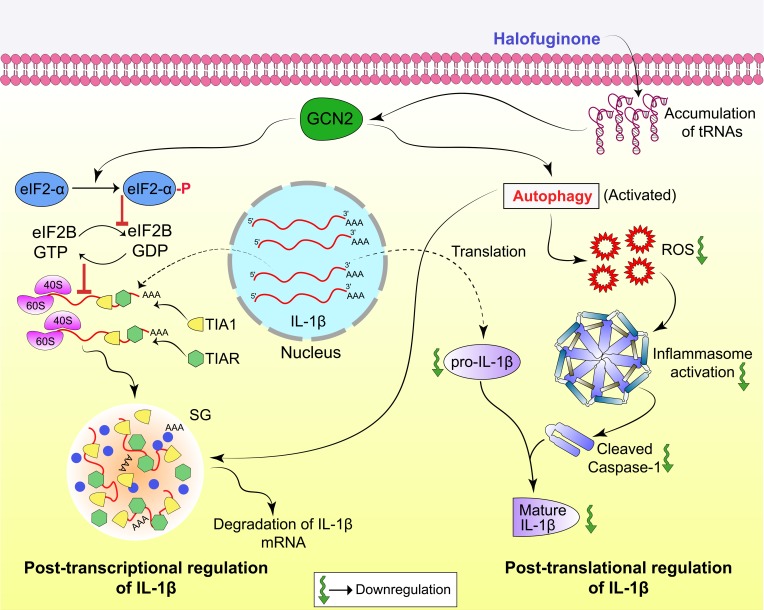
Proposed model of the mechanism by which HF suppresses IL-1β expression. HF induces activation of the amino acid starvation sensor, GCN2, which triggers SG formation and autophagy. SG formation regulates IL-1β expression at mRNA level via translational silencers, TIA-1/TIAR and RBPs. Later, these SGs are cleared by autophagy. On the other hand, the activated autophagy process decreases ROS levels, thereby inhibiting inflammasome activation, leading to decrease in active caspase-1 and secretion of mature IL-1β. eIF2, eukaryotic initiation factor 2; GCN2, general control nonderepressible 2 kinase; GDP, guanosine diphosphate; GTP, guanosine triphosphate; HF, Halofuginone; IL-1β, interleukin 1β; RBP, RNA-binding protein; ROS, reactive oxygen species; SG, stress granule; TIA-1, T cell–restricted intracellular antigen-1; TIAR, TIA-1–related.

It is well known that HF activates GCN2 by acting competitively with proline, thereby inhibiting prolyl-tRNA synthetase activity of EPRS, known to participate in the interferon γ (IFNγ)-activated inhibitor of translation complex (GAIT) [14]. Activation of the GAIT complex by IFNγ results in the suppression of proinflammatory gene expression through the binding of the GAIT complex to the 3’UTRs of inflammatory cytokines [43]. However, LPS-induced IL-1β production is not impaired in PBMCs incubated with IFNγ [44], indicating stress-specific regulation of IL-1β. Our findings indicate that HF-induced eIF2-α phosphorylation triggers riboclustering or SG formation, an important PTR event in the regulation of inflammatory cytokines such as IL-1β [7]. Previous studies suggest that cytokine mRNAs, including IL-1β, bear ARE sequences at 3’UTR [45], which confer tight post-transcriptional regulation mainly mediated by stress-driven activation of ISR pathways, resulting in translational blockade [7]. Translationally silenced mRNAs trigger ribocluster or SG formation via recruitment of RBPs, which in turn dictate the mRNA’s stability/decay [23]. The existence of such regulatory mechanisms assist the cells in turning “on” or “off” its protein synthesis machinery as per cellular requirements to overcome stressful conditions like amino acid starvation [7]. Our findings reveal that HF-induced GCN2 activation causes translational silencing of IL-1β mRNA through enhanced expression of translation silencer RBPs, TIA-1 and TIAR, which in turn assists cytoplasmic repositioning of the IL-1β mRNA transcript from polysomes to SGs (Fig 7). Previous studies have shown that the eukaryotic SGs are cleared by autophagy [27]. In line with the earlier observations, our findings also reveal that HF induces autophagy and facilitates the post-transcriptional degradation of SG-bound IL-1β mRNAs. Furthermore, autophagy could also influence mature IL-1β production post-translationally through ROS-dependent inhibition of inflammasome activation [46]. Our results demonstrate that HF-induced autophagy suppresses ROS production, thereby inhibiting active IL-1β production post-translationally. These findings highlight the centrality of autophagy in controlling post-transcriptional as well as post-translational regulatory events.

Exacerbated expression of IL-1β is associated with the development of IBDs such as colitis [36], and pharmacological neutralization of these cytokines reduces the severity of colitis [47]. Meta-analysis of publicly available UC and CD gene expression data revealed a substantial reduction of GCN2 expression in the PBMCs. Our data indicate that HF significantly reduces serum IL-1β levels and the inflammatory pathology in a murine model of DSS-induced colitis and highlight the therapeutic potential of HF-induced GCN2–AAR pathways in controlling intestinal inflammation. Altogether, this study reveals a novel mechanism of IL-1β regulation and further suggests that pharmacological activation of the evolutionarily conserved cytoprotective AAR pathway might offer an effective therapeutic intervention against inflammatory diseases.

## Materials and methods

### Ethics statement

The institutional animal care and use committee of the University of Hyderabad approved all the animal experiments. The study approval number is IAEC/UH/151/2017/01/NK/P21/Mice C57BL/6 or BALB/c/M-54.

### Cells, reagents, and plasmids

DR-Wildtype (ATCC CRL2977) and GCN2-KO-DR (ATCC CRL2978) MEFs, purchased from American Type Culture Collection (ATCC), were maintained in DMEM containing 10% (vol/vol) FBS (Invitrogen), 100 units/ml penicillin, 100 μg/ml streptomycin, 2 mM L-Glutamine, 1 mM sodium pyruvate, and 0.05 mM 2-mercaptomethanol. Sodium thioglycolate (Sigma-Aldrich) was used to generate thioglycolate-elicited peritoneal macrophages. LPS (Sigma-Aldrich) and ATP (Sigma-Aldrich) were used at a concentration of 500ng/ml and 5mM, respectively, to stimulate BMDMs, peritoneal macrophages, or J774A.1 cells cultured in DMEM containing 10% (vol/vol) FBS (Invitrogen), 100 units/ml penicillin, 100 μg/ml streptomycin, 2 mM L-Glutamine, and 1 mM sodium pyruvate. HF hydrobromide (*trans*-[±]-7-Bromo-6-chloro-3-[3-(3-hydroxy-2-piperidinyl)-2-oxopropyl]-4[3*H*])quinazolinonemonohydrobromide (Sigma-Aldrich); 3-MA (Sigma-Aldrich), and Wortmannin (Sigma-Aldrich) were used at a concentration of 5 mM or 5 μM. Cycloheximide and Act-D (Sigma-Aldrich) were used at 1 μg/ml and 10 μg/ml. Rapamycin (Sigma-Aldrich) was used at a concentration of 200 nM. Lipofectamine 3000 was from Life Technologies (L3000015) and Sepharose G beads from Sigma-Aldrich. pCMV6 IL-1β was purchased from Origene.

### Generation of murine BMDMs

Bone marrow from femurs and tibiae of C57BL/6J mice were flushed with ice-cold PBS/RPMI and cultured at a density of 5 × 10^6^ to 7 × 10^6^ cells in 10-cm Petri dishes in DMEM medium with 10% (v/v) FBS, 100 units/ml penicillin, 100 μg/ml streptomycin, 2 mM L-Glutamine, and 1 mM sodium pyruvate (Invitrogen), in the presence of M-CSF (20 ng/ml, Peprotech). Fresh medium supplemented with fresh M-CSF was added to the cultures on d 4. BMDMs were harvested on d 6 for further experiments.

### Immunoblotting

Cells were washed with ice-cold 1X PBS followed by lysis in lysis buffer (20 mM HEPES, pH 7.5, 150 mM NaCl, 1% Triton-X 100, 10% Glycerol, 1 mM EDTA, 100 mM NaF, 17.5 mM β-glycerophosphate, 1 mM phenyl methyl sulphonyl fluoride, 4 mg/ml aprotinin, and 2 mg/ml pepstatin), supplemented with protease inhibitor cocktail tablets (Sigma-Aldrich) and incubated on ice for 30 min with occasional vortexing as described earlier [17][48]. The lysates were cleared by centrifugation at 12,000 rpm for 15 min at 4°C. Protein concentration was quantified using BCA protein assay kit (71285–3; Novagen), and equal concentrations of proteins were loaded on SDS-PAGE (12%–15% Tris-glycine gel) and the separated proteins transferred onto nitrocellulose membrane (S80209, Pall Corporation) by electroblotting. The membranes were blocked with 5% skimmed milk (Hi-Media). For detection of eIF2α-P, GCN2-P, 5% bovine serum albumin (Sigma-Aldrich) in 1XPBS was used to block the membranes. The antibodies used for immunoblot analysis were rabbit anti–eIF2α-P, anti–GCN2-P (Cell Signaling Technology), rabbit anti–IL-1β (Santa Cruz), rabbit anti pro–caspase-1 (Santa Cruz), goat anti–cleaved caspase-1 (Santa Cruz), rabbit anti–β-actin (Sigma-Aldrich), goat anti–TIA-1/TIAR (Santa Cruz), rabbit anti-LC3 (Cell Signaling Technology), and mouse anti-Flag (Sigma-Aldrich). After overnight incubation at 4°C with appropriate primary antibodies, the membranes were washed thrice with 1X TBST to remove nonspecifically bound antibodies and probed with appropriate secondary antibodies. Protein bands were visualized with Super Signal ECL-prime chemiluminescent substrate (RPN2232; GE Healthcare) and chemiluminescence captured on X-ray films or ChemiDoc Touch Imaging System (BioRad). Densitometry analysis of protein expression was done using NIH Image J software (National Institute of Health).

### Measurement of mature IL-1β and cleaved caspase-1

BMDMs or J774A.1 cells cultured in OPTI-MEM media (51985034; Gibco) were primed with LPS followed by stimulation with or without HF for 6 h. Following treatment, culture supernatants were harvested and subjected to methanol-chloroform precipitation as described previously [49]. Briefly, 500 μl of culture supernatant was mixed with 500 μl of methanol followed by the addition of 100 μl of chloroform, vortexed, and centrifuged at 13,000 rpm for 5 min; the aqueous layer was removed, and again, 500 μl methanol was added, vortexed, and centrifuged at 14,000 rpm for 2 min. Later, supernatants were removed, and the pellet was dried by heating at 50°C for 5 min and dissolved in 50 μl of 2X sample buffer; this was followed by boiling at 95°C for 5 min before separation on 12% SDS-PAGE (Tris-glycine gel). Proteins were transferred onto nitrocellulose membrane by electroblotting, and the expression levels of cleaved caspase-1 and mature IL-1β were analyzed by immunoblotting as described earlier [49].

### qRT-PCR

Total RNA was isolated using TRIZOL Reagent (Invitrogen). A total of 1,000 ng of RNA was reverse transcribed into cDNA using Verso cDNA Synthesis Kit (Thermo Scientific) according to manufacturer’s instructions. qRT-PCR was performed using Master cycler ep real plex (Eppendorf). A total of 50 ng cDNA was amplified using Syber Green mix (Kappa Biosystems) with gene-specific primers as described (S1 Table). The relative mRNA expression of each gene were normalized to the housekeeping gene GAPDH/β-actin as described earlier [16].

### ELISA

Cytokine levels following HF treatment in the LPS-primed macrophages were measured in the culture supernatants by two-site sandwich ELISA as described earlier [16]. ELISA was performed using kits (BD OptEIA) obtained from BD Biosciences following the manufacturer’s instructions.

### Immunofluorescence microscopy

Cells were allowed to grow overnight on coverslips at low density and stimulated with appropriate treatments. Later, these cells were washed and fixed with 4% paraformaldehyde in 1X PBS and permeabilized with 0.2% Triton-X-100 (T8787; Sigma-Aldrich). Nonspecific binding was blocked with 5% BSA (Calbiochem) for 1 h at RT, followed by incubation with anti–TIA-1/TIAR antibodies (Sc-1751 and Sc-1749; Santa Cruz) for the visualization of SGs—or anti-LC3 antibodies (4108; Cell Signaling) for the visualization of autophagosomes for 2 h at RT followed by incubation with suitable secondary antibodies for 2 h at RT (Alexa Flour 488 and Alexa Flour 555 [Invitrogen]). Immunostained cells were finally mounted using Prolong Gold antifade reagent containing DAPI (Invitrogen). Immunofluorescence signals were detected using a LSM510 confocal microscope (Zeiss), and images were captured and analyzed using the Zeiss LSM Image Browser.

### RIP

IP was carried out to assess the association of IL-1β with RBPs such as TIA-1 and TIAR. RBP–IL-1β–mRNA complexes were immune precipitated as described previously [50]. Briefly, 50 ×10^6^cells were primed with LPS followed by treatment with and without HF. Following treatment, cells were harvested, and cell lysates were prepared using polysome lysis buffer (100 mM KCl, 5 mM MgCl2, 10 mM Hepes, pH 7.0, and 0.5% NP-40 including 1 mM DTT, 200 μM heparin, 0.2% vanadyl ribonucleoside complexes, 0.2 mM PMSF, 1 mg/ml pepstatin A, 5 mg/ml bestatin, and 20 mg/ml leupeptin). For IP, 50 μl of beads were incubated overnight at 4°C with 10 μg of either goat IgG or anti–TIA-1/TIAR antibodies (Santa Cruz). Furthermore, the antibody-coated protein A/G beads were extensively washed with NT2 buffer (50 mM Tris, pH7.4, 150 mM NaCl, 1 mM MgCl2, 0.05% NP-40) 4 to 5 times, followed by resuspension in 500 μl of NT2 buffer containing 200 μM heparin, 5 μl of vanadyl ribonucleoside complexes, 10 μl of a 100 mM DTT stock, and 20 mM EDTA. The beads were incubated with cell lysates for 4 h at 4°C with continuous rotation. Following incubation, the beads–antibody–mRNP complexes were washed at least 5 times with ice-cold NT2 buffer and resuspended in 100 μl NT2 buffer supplemented with 0.1% SDS and 30 μg proteinase K, followed by 30-min incubation at 55°C in water bath. After digestion of protein in IP material, RNA was extracted using TRIZOL reagent (Invitrogen), which was further used to perform RT-PCR for examining IL-1β mRNA transcripts in the pull-down extracts. RT-PCR was carried out using IL-1β–specific primers with the following thermal cycler parameters:1 cycle of 94°C for 2 min followed by 30 cycles of 30-s denaturation at 94°C, 30-s annealing at 56°C, 40-s extension at 72°C, and 5-min final extension at 72°C. PCR products were visualized after electrophoresis in 1.5% agarose, ethidium bromide–stained gels. The levels of IL-1β mRNA in IP were also determined by qRT-PCR and normalized to GAPDH mRNA levels and control goat IgG IP following the formula as mentioned previously [51]. Fold enrichment = 2^−ddCt^, where ddCt = ([Ct _IL-1β mRNA, IP_—Ct _GAPDH, IP_]–[Ct _IL-1β mRNA, Goat IgG IP_—Ct _GAPDH, Goat IgG IP_]).

### Site-directed mutagenesis for deletion of ARE sequence in IL-1β mRNA 3’UTR

pCMV6 IL-1β plasmid (Origene, Rockville, MD) served as the template to design the 3 sets of primers lacking 3 stretches of ARE sequences (TATTTATTTAT, TATTTATT, and TATTTA) in IL-1β 3’UTR. Three different sets of primers were designed to amplify the entire sequence of the plasmid except the 3 regions enriched with AT sequences to be deleted. The primers were designed in such a manner that the first mutant plasmid serves as a template to generate the second mutant. Likewise, the second mutant serves as a template for the third deletion mutant (S2 Table). The 3 oligonucleotide-based deletions were carried out using 2.5U of Phusion DNA polymerase (NEB Labs) in a 50 μl PCR containing 50 ng of source plasmid, 125 ng of forward and reverse primer each, and 300 μM of dNTP mix. The reaction was carried out with 18-cycle PCR using a 3-step PCR method with conditions 98°C for 30 s, 98°C for 30 s, 55 to 60°C for 1 min, 68°C for 7 min, and 72°C for 10 min. Subsequently, *Dpn* I was added to PCR reaction and then incubated for 1 h at 37°C to remove the native plasmid from PCR reaction followed by transformation into XL1-Blue cells. Three colonies were randomly picked for every mutant and grown overnight in a shaking incubator at 37°C. Consequently, plasmids were extracted from the grown culture using “Gene JET Plasmid Miniprep Kit” as per the manufacturer’s instructions (ThermoFisher Scientific) and then subjected to DNA sequencing to confirm the oligonucleotide-directed mutagenesis. The deletion of the 3 DNA stretches TATTTATTTAT, TATTTATT, and TATTTA from the wild *Mus musculus* IL-1β gene sequence when compared to the source gene sequence of the *Mus musculus* IL-1β gene sequence was confirmed using the online alignment program CLUSTAL Omega. The final alignment file was generated using the online tool ESPript 3.0 (http://espript.ibcp.fr/ESPript/cgi-bin/ESPript.cgi).

### Transient transfections

Invitro transient transfection was carried out using Lipofectamine 3000 reagent according to manufacturer’s instructions. Briefly, HEK293T or J774A.1 cells were transfected at 70% confluency in reduced-serum media OptiMEM (Gibco, Life Technologies) with plasmid constructs pCMV6-IL-1β (Origene, Rockville), pcDNA3-GCN2, pEYFP-TIA-1, pEYFP-TIAR, or vector controls. After 6 h, media were replaced with complete DMEM. Functional analysis was performed at 24 to 48 h post transfection.

### RNA interference

The smart pool siRNA (40 picomoles) targeting mouse GCN2, TIAR, or control siRNA with scrambled sequences (Dharmacon)—or TIA-1, PKR, ATG16L1, and eIF2-α (Eurogentec)—were introduced in J774A.1 macrophages by Lipofectamine 3000 reagent as described earlier [52]. After 24 h, cells were stimulated with or without HF. Cells and culture supernatants were harvested for further assays, including ELISA or immunoblotting, as described above.

### ROS estimation

For examination of ROS levels, LPS-stimulated macrophages were treated with HF (Sigma-Aldrich) or MAZ1310 for 6 h. Following treatment, cells were harvested and incubated with CM-H2DCFDA (I36007; Life Technologies) for 30 min at 37°C in FACS buffer as described earlier [48]. Later, these cells were washed extensively with HBSS and percent fluorescent cells analyzed by flow cytometry. For mitochondrial ROS estimation, cells stimulated with LPS plus ATP or cells stimulated with LPS plus ATP plus HF were stained using MitoSOX (M36008, Life Technologies) dye for 15 min (manufacturer’s instructions) and analyzed by flow cytometry.

### Gene expression meta-analysis of UC and CD

Raw data of 3 UC data sets (D1-GSE9686, D2-GSE10191, and D-3 GSE10616) [53–55] were retrieved from the GEO database. An additional data set (GSE11499) [56] including both UC and CD gene expression data was curated into 2 custom data sets (D4, D5) based on the type of disease. Sample sources included colon tissue (D1, D2, and D3) and PBMCs (D4, D5) (sample information is detailed in S3 Table). In the case of the absence of healthy specimens, expression data were downloaded from another data set [57] available on the GEO database. The 5 data sets were devised and assigned for individual analysis. Raw data were preprocessed using RMA normalization method. Our analysis of interest included UC versus healthy data sets (D1, D2, D3, and D4) and CD versus healthy (D5). A student *t* test was conducted to check for differential expression in each gene. Genes with *p*-values less than 0.05 were considered differentially expressed genes (DEGs). Analyses were performed in R using various Bioconductor packages [58–60]. After the analyses, common DEGs from the 2 sets of DEGs were extracted. A total of 4,038 genes were commonly expressed across the 5 data sets.

### DSS-induced colitis

C57BL/6J mice were treated with intraperitoneal injection of 0.2 mg/kg [17] HF hydrobromide (Sigma-Aldrich), reconstituted in DMSO, and diluted in PBS for 3 d prior to DSS administration as described earlier [2]. Acute colitis was induced in mice by adding 5% DSS (MW36,000–50,000; MP Biomedicals) in drinking water for 7 d followed by normal drinking water for 3 d [4]. Mice were euthanized on d 10; colon tissues were examined for histological analysis, and IL-1β expression was analyzed in tissue lysates by immunoblotting.

### Histology

Colons were excised from the euthanized mice and prepared for histological analysis by the “Swiss roll technique” as described earlier [61], and tissues embedded in OCT media were sectioned and stained with hematoxylin–eosin (HE) staining.

### Statistical analysis

All data were represented as mean ± SEM of 1 of 3 separate experiments. To assess the significance of the difference between groups, a two-sample, unpaired student *t* test was executed using Graph Pad Prism software.

## Supporting information

S1 Fig HF is not toxic to cells at concentrations below 40 nM. Mouse macrophages primed with LPS or left unstimulated were cultured with HF at different concentrations as indicated for 6 h, 12 h, and 24 h. Percent viable cells were assayed by using MTT (S1 Data). Data are representative of 1 of 3 independent experiments. HF, Halofuginone; MTT, 3-(4,5-dimethylthiazol-2-yl)-2,5- diphenyltetrazolium bromide.
(TIF)

S2 Fig HF suppresses IL-1β production in LPS-primed macrophages. (A) Mouse BMDMs were primed with LPS (500 ng/ml), followed by HF stimulation for the indicated times; ATP (5mM) was added to the cultures for 30 min prior to harvest. IL-1β levels were measured by ELISA (S1 Data). ***P <0.001 (two-way ANOVA and Bonferroni post-test). (B) Analysis of production of proinflammatory cytokines IL-1β and TNF-α from BMDMs stimulated with LPS or LPS plus HF by ELISA. No ATP was added to the cultures (S1 Data). *P < 0.05, **P < 0.01. (C) Analysis of production of proinflammatory cytokines TNF-α and IL-6 from BMDMs stimulated with LPS or LPS plus HF by ELISA. ATP 5 mM was added to the cultures (S1 Data). *P < 0.05, **P< 0.01. (D, E) Analysis of levels of proinflammatory cytokines IL-1β, TNF-α, and IL-6 in culture supernatants of peritoneal macrophages by ELISA (panel D) or J774A.1 macrophages (panel E) primed with LPS followed by HF stimulation. ATP (5 mM) was added to the LPS-stimulated cultures for 30 min at the end of the experiment (S1 Data). *P < 0.05, **P < 0.005, ***P < 0.0005. (F) Analysis of expression of proinflammatory cytokine IL-1β by ELISA in culture supernatants of BMDMs stimulated with various TLR ligands (S1 Data). (G) Immunoblot analysis of pro–IL-1β expression in the lysates of BMDMs stimulated with LPS or LPS plus HF; β- actin was used as loading control. **P < 0.005. Error bars indicate mean ± SEM. Data represent 1 experiment of 3 independent experiments. BMDM, bone marrow–derived macrophage; HF, Halofuginone; IL-1β, interleukin 1β; LPS, lipopolysaccharide; TLR, toll-like receptor; TNF-α, tumor necrosis factor α.
(TIF)

S3 Fig HF reduces IL-1β and IL-18 mRNA levels. (A) qRT-PCR analysis of proinflammatory cytokines IL-18, TNF-α, and IL-6 in BMDMs stimulated with LPS or LPS plus HF (S1 Data). (B) IL-1β mRNA levels in HEK293T cells transfected with pCMV6-IL-1β for 36 h followed by treatment with or without Act-D (2 h) and further treatment with HF (20 nM) (S1 Data). *P < 0.05, **P < 0.01. (C) qRT-PCR analysis of IL-1β mRNA expression in LPS-primed macrophages treated with HF in presence or absence of cycloheximide (S1 Data). Error bars indicate mean ± SEM. Data are representative of 1 of 3 separate experiments. Act-D, actinomycin-D; BMDM, bone marrow–derived macrophage; HEK293T, human embryonic kidney cells 293T; HF, Halofuginone; IL-1β, interleukin 1β; LPS, lipopolysaccharide; qRT-PCR, quantitative reverse transcription PCR; TNF-α, tumor necrosis factor α.
(TIF)

S4 Fig HF induces SG formation in BMDMs. Immunofluorescence imaging of SGs (indicated by white arrows) in BMDMs left untreated (media) or treated with HF (20 nM) at time points specified. Scale bars, 5μm. Data are representative of 1 of 4 independent experiments. BMDM, bone marrow–derived macrophage; HF, Halofuginone; SG, stress granule.
(TIF)

S5 Fig Decreased GCN2–eIF2 signaling and RBPs (TIA-1/TIAR) in macrophages result in increased IL-1β production in response to LPS stimulation, and reduced responsiveness to HF treatment. (A) J774A.1 macrophages were transfected with control siRNA- or GCN2-specific siRNA. After 24 h, cell lysates were prepared and analyzed for GCN2 expression by western blotting. (B) Densitometric analysis of western blot (shown in main Fig 2H). The levels of pro and mature IL-1β was normalized to endogenous control β-Actin (S1 Data). (C) qRT-PCR analysis of IL-1β and TNF-α mRNA expression in control or GCN2-silenced macrophages primed with LPS and further treated or untreated with HF (20 nM) (S1 Data). (D) Measurement of IL-1β levels in the culture supernatants of control or eIF2-α siRNA–silenced macrophages treated with LPS or LPS plus HF by ELISA. ATP was added for 30 min at the end of the experiment (S1 Data). (E) Immunoblot analysis of TIA-1/TIAR in HF (20 nM)-, LPS-, or LPS plus HF–treated J774A.1 macrophages. β-actin used as a loading control. (F) qRT-PCR analysis of IL-1β and TNF-α mRNA expression in control or TIAR-silenced J774A.1 macrophages primed with LPS and further treated or untreated with HF (20 nM) (S1 Data). (G) Densitometric analysis of TIA-1 and TIAR expression levels in control siRNA or TIA-1/TIAR siRNA–transfected J774A.1 macrophages. The TIA-1/TIAR levels were normalized to endogenous control β-actin, and percent inhibition in TIA-1/TIAR–silenced macrophages over control was represented in the graph (S1 Data). (H) Immunoblot analysis of TIA-1 in control or eIF2-α–silenced macrophages treated with HF as indicated. β-actin was used as loading control. *P < 0.05. Error bars indicate mean ± SEM. Data represent 1 experiment of 3 independent experiments. eIF2, eukaryotic initiation factor 2; GCN2, general control nonderepressible 2 kinase; HF, Halofuginone; IL-1β, interleukin 1β; LPS, lipopolysaccharide; qRT-PCR, quantitative reverse transcription PCR; RBP, RNA-binding protein; siRNA, small interfering RNA; TIA-1, T cell–restricted intracellular antigen-1; TIAR, TIA-1–related; TNF-α, tumor necrosis factor α.
(TIF)

S6 Fig HF-induced effect on IL-1β production is independent of PKR. IL-1β protein levels in the culture supernatant of control siRNA–or PKR siRNA–transfected J774A.1 macrophages, stimulated with LPS (500 ng/ml) followed by stimulation with different concentrations of HF. ATP (5 mM) was added to the cultures for 30 min at the end of the experiment (S1 Data). HF, Halofuginone; IL-1β, interleukin 1β; LPS, lipopolysaccharide; PKR, protein kinase R; siRNA, small interfering RNA.
(TIF)

S7 Fig LPS-induced TNF-α but not IL-6 transcripts are targeted by the RBPs TIA-1/TIAR during HF treatment. qRT-PCR analysis of IL-6 and TNF-α mRNA in RIP (pull-down using TIA-1/TIAR or IgG) of LPS-primed J774A.1 macrophages untreated or treated with HF (20 nM) (S1 Data). *P < 0.05. Error bars indicate mean ± SEM. Data represent 1 experiment of 3 independent experiments. HF, Halofuginone; IgG, immunoglobulin G; IL-6, interleukin 6; LPS, lipopolysaccharide; qRT-PCR, quantitative reverse transcription PCR; RBP, RNA-binding protein; TIA-1, T cell–restricted intracellular antigen-1; TIAR, TIA-1–related; TNF-α, tumor necrosis factor α.
(TIF)

S8 Fig HF promotes induction of autophagy. (A) LPS-primed or -unprimed J774A.1 macrophages were treated with HF for 6 h in presence or absence of autophagy inhibitor CQ; lysates were assayed for the expression of autophagy proteins LC3-I and LC3-II by immunoblotting. β-actin was used as loading control. (B) p62 expression in macrophages treated with HF in presence of lysosomal inhibitor, CQ. β-actin was used as loading control. (C) qRT-PCR analysis of p62 mRNA in macrophages treated with HF as indicated (S1 Data). (D) qRT-PCR analysis of p62 mRNA in WT or GCN2^−/−^macrophages treated with HF (S1 Data). (E) HEK293T cells were transiently transfected with vector or GCN2. After 24 h, cells were starved for indicated time points or treated with chloroquine. Lysates were analyzed for LC3 protein conversion by using immunoblotting. β–actin was used as loading control (S1 Data, right panel). GCN2, general control nonderepressible 2 kinase; HEK293T, human embryonic kidney cells 293T; HF, Halofuginone; LC3, microtubule-associated protein 1A/1B light chain 3; qRT-PCR, quantitative reverse transcription PCR; WT, wild-type.
(TIF)

S9 Fig HF-induced autophagy controls IL-1β production by targeting SGs for clearance and by controlling mitochondrial ROS. (A) Immunofluorescence microscopy imaging of SGs (stained by TIA-1/TIAR) colocalization with LC3 during HF treatment. Nuclei were stained with DAPI; scale bars, 5 μM. (B) Levels of IL-1β protein in the culture supernatant of control siRNA or Atg16L1 siRNA–transfected J774A.1 macrophages. Macrophages were LPS (500 ng/ml)-primed followed by stimulation with HF (20 nM). ATP (5 mM) was added for 30 min at the end of the experiment (S1 Data). (C, D) Detection of mitochondrial ROS in LPS-primed macrophages stimulated with different concentrations of HF or control MAZ1310 followed by mitochondrial ROS estimation using MitoSOX dye through flow cytometry (panel C), and colorimetric fluorescence measurement (panel D) (S1 Data). *P < 0.05, **P < 0.005. Error bars indicate mean ± SEM. Data represent 1 of 3 independent experiments. HF, Halofuginone; IL-1β, interleukin 1β; LPS, lipopolysaccharide; ROS, reactive oxygen species; SG, stress granule; siRNA, small interfering RNA; TIA-1, T cell–restricted intracellular antigen-1; TIAR, TIA-1–related.
(TIF)

S10 Fig Meta-analysis of gene expression data sets reveals differential expression of GCN2 in PBMCs and colon samples from patients with UC and CD. (A) Pipeline of individual gene expression data set analysis followed by meta-analysis. (B) Heat map (left) representing 4,038 DEGs that were commonly expressed across the 5 data sets, in which GCN2 is down-regulated in PBMCs and up-regulated in colon data sets (right heat map). (C) Box-plots representing change in normalized intensity values of GCN2 between diseased (UC and CD) and healthy controls (S1 Data). CD, Crohn’s disease; DEG, differentially expressed gene; GCN2, general control nonderepressible 2 kinase; PBMC, peripheral blood mononuclear cell; UC, ulcerative colitis.
(TIF)

S1 Table List of mouse gene-specific primers. The primer sequences listed in the table were used to examine the analysis of gene expression of various genes.
(XLSX)

S2 Table List of primers used for site-directed mutagenesis. Three different sets of primers given in the table were designed to amplify the entire sequence of the pCMV6-IL-1β plasmid except the 3 regions enriched with AT sequences to be deleted. The primers were designed in such a way that the first mutant plasmid serves as a template to generate the second mutant. Likewise, the second mutant serves as a template for the third deletion mutant.
(XLSX)

S3 Table GEO data sets used for the meta-analysis study. The table summarizes information of the GEO data sets containing UC and CD patient samples. The table also includes the sample source and the number of control samples. CD, Crohn’s disease; GEO, Gene Expression Omnibus; UC, ulcerative colitis.
(XLSX)

S1 Data (XLSX)

## Abbreviations

3-MA: 3-methyl adenine
AAR: amino acid starvation response
Act-D: actinomycin-D
ALU: aluminium hydroxide
ATCC: American Type Culture Collection
Atg16L1: autophagy-related 16-like 1
Atg7: autophagy-related protein 7
BMDM: bone marrow–derived macrophage
CD: Crohn’s disease
CMV: cytomegalovirus
CQ: chloroquine
DAMP: damage-associated molecular pattern
DEG: differentially expressed gene
dsRNA: double-stranded RNA
DSS: dextran sulfate sodium
EAE: experimental autoimmune encephalomyelitis
eIF2: eukaryotic initiation factor 2
EPRS: glutamyl-prolyl-tRNA synthetase
GAIT: IFNγ-activated inhibitor of translation complex
GCN2: general control nonderepressible 2 kinase
GEO: Gene Expression Omnibus
GTP: guanosine triphosphate
HE: hematoxylin–eosin
HEK293T: human embryonic kidney cells 293T
HF: Halofuginone
HRI: heme-regulated eIF2α kinase
IBD: inflammatory bowel disease
IFNγ: interferon γ
IgG: immunoglobulin G
IL-1β: interleukin 1β
IP: immunoprecipitation
ISR: integrated stress response
LC3: microtubule-associated protein 1A/1B light chain 3
LPS: lipopolysaccharide
MAZ1310: (2R,3S)-2-[3-(7-Bromo-6-chloro-4-oxo-3(4H)-quinazolinyl)-2-oxopropyl]-3-hydroxy-1-piperidinecarboxylate de 2-methyl-2-propanyle
MEF: mouse embryonic fibroblast cells
MOI: multiplicity of infection
MSU: monosodium urate
mTOR: mammalian target of rapamycin
PBMC: peripheral blood mononuclear cell
PKR: protein kinase R
PERK: RNA-dependent protein kinase-like endoplasmic reticulum kinase
PTR: post-transcriptional reprogramming
qRT-PCR: quantitative reverse transcription PCR
RBP: RNA-binding protein
RIP: RNA immunoprecipitation
ROS: reactive oxygen species
p62/SQSTM1: sequestosome 1
Ser51: serine 51
SG: stress granule
siRNA: small interfering RNA
Th17: T helper 17 cells
TIA-1: T cell–restricted intracellular antigen-1
TIAR: TIA-1–related
TLR: toll-like receptor
TNF-α: tumor necrosis factor α
tRNA: transfer RNA
UC: ulcerative colitis
WT: wild-type
