# Supplementary figures and images for: Amino acid starvation sensing dampens IL-1β production by activating riboclustering and autophagy
Source: PLoS Biol. 2018 Apr 5;16(4):e2005317. doi: 10.1371/journal.pbio.2005317 (PMC5903674; doi:10.1371/journal.pbio.2005317)

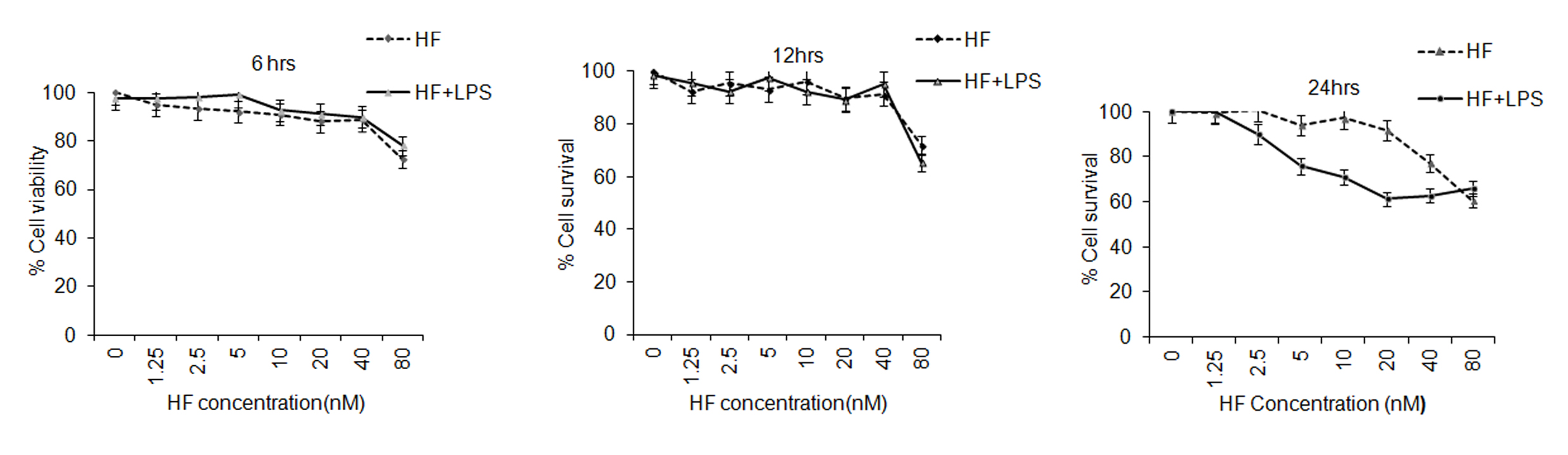

Supplement: S1 Fig — Mouse macrophages primed with LPS or left unstimulated were cultured with HF at different concentrations as indicated for 6 h, 12 h, and 24 h. Percent viable cells were assayed by using MTT (S1 Data). Data are representative of 1 of 3 independent experiments. HF, Halofuginone; MTT, 3-(4,5-dimethylthiazol-2-yl)-2,5- diphenyltetrazolium bromide. (TIF) [file pbio.2005317.s001.tif]

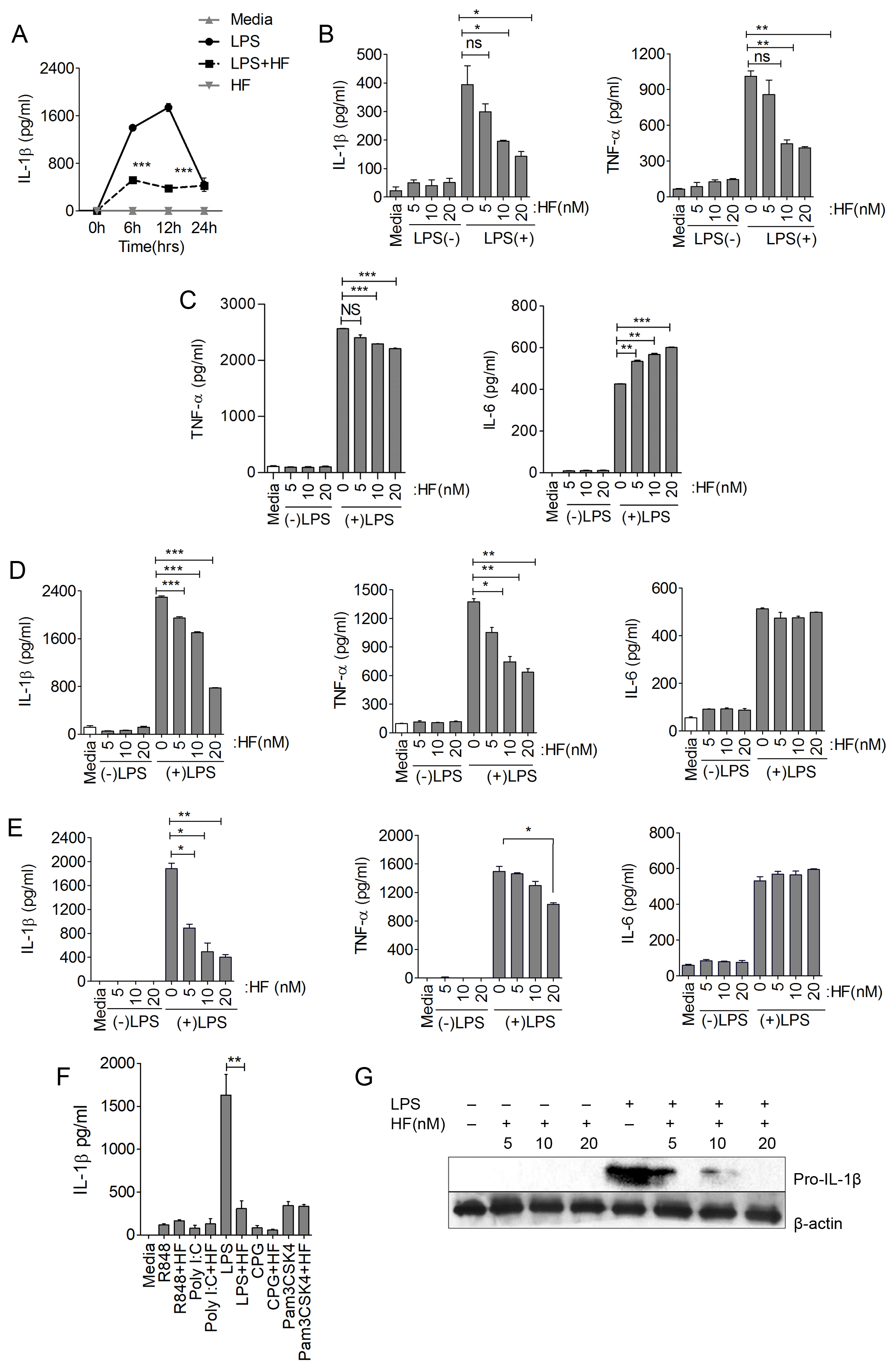

Supplement: S2 Fig — (A) Mouse BMDMs were primed with LPS (500 ng/ml), followed by HF stimulation for the indicated times; ATP (5mM) was added to the cultures for 30 min prior to harvest. IL-1β levels were measured by ELISA (S1 Data). ***P <0.001 (two-way ANOVA and Bonferroni post-test). (B) Analysis of production of proinflammatory cytokines IL-1β and TNF-α from BMDMs stimulated with LPS or LPS plus HF by ELISA. No ATP was added to the cultures (S1 Data). *P < 0.05, **P < 0.01. (C) Analysis of production of proinflammatory cytokines TNF-α and IL-6 from BMDMs stimulated with LPS or LPS plus HF by ELISA. ATP 5 mM was added to the cultures (S1 Data). *P < 0.05, **P< 0.01. (D, E) Analysis of levels of proinflammatory cytokines IL-1β, TNF-α, and IL-6 in culture supernatants of peritoneal macrophages by ELISA (panel D) or J774A.1 macrophages (panel E) primed with LPS followed by HF stimulation. ATP (5 mM) was added to the LPS-stimulated cultures for 30 min at the end of the experiment (S1 Data). *P < 0.05, **P < 0.005, ***P < 0.0005. (F) Analysis of expression of proinflammatory cytokine IL-1β by ELISA in culture supernatants of BMDMs stimulated with various TLR ligands (S1 Data). (G) Immunoblot analysis of pro–IL-1β expression in the lysates of BMDMs stimulated with LPS or LPS plus HF; β- actin was used as loading control. **P < 0.005. Error bars indicate mean ± SEM. Data represent 1 experiment of 3 independent experiments. BMDM, bone marrow–derived macrophage; HF, Halofuginone; IL-1β, interleukin 1β; LPS, lipopolysaccharide; TLR, toll-like receptor; TNF-α, tumor necrosis factor α. (TIF) [file pbio.2005317.s002.tif]

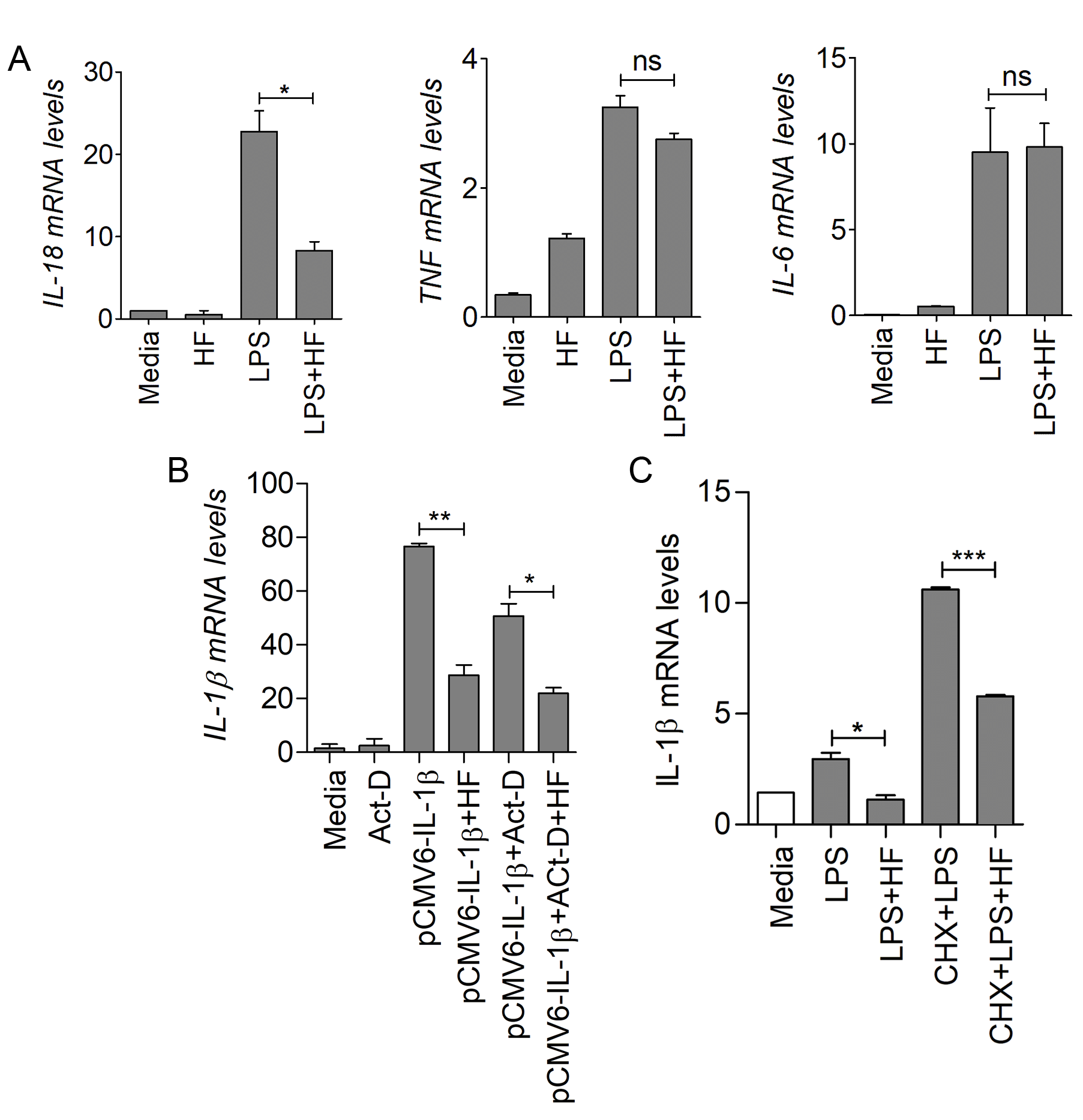

Supplement: S3 Fig — (A) qRT-PCR analysis of proinflammatory cytokines IL-18, TNF-α, and IL-6 in BMDMs stimulated with LPS or LPS plus HF (S1 Data). (B) IL-1β mRNA levels in HEK293T cells transfected with pCMV6-IL-1β for 36 h followed by treatment with or without Act-D (2 h) and further treatment with HF (20 nM) (S1 Data). *P < 0.05, **P < 0.01. (C) qRT-PCR analysis of IL-1β mRNA expression in LPS-primed macrophages treated with HF in presence or absence of cycloheximide (S1 Data). Error bars indicate mean ± SEM. Data are representative of 1 of 3 separate experiments. Act-D, actinomycin-D; BMDM, bone marrow–derived macrophage; HEK293T, human embryonic kidney cells 293T; HF, Halofuginone; IL-1β, interleukin 1β; LPS, lipopolysaccharide; qRT-PCR, quantitative reverse transcription PCR; TNF-α, tumor necrosis factor α. (TIF) [file pbio.2005317.s003.tif]

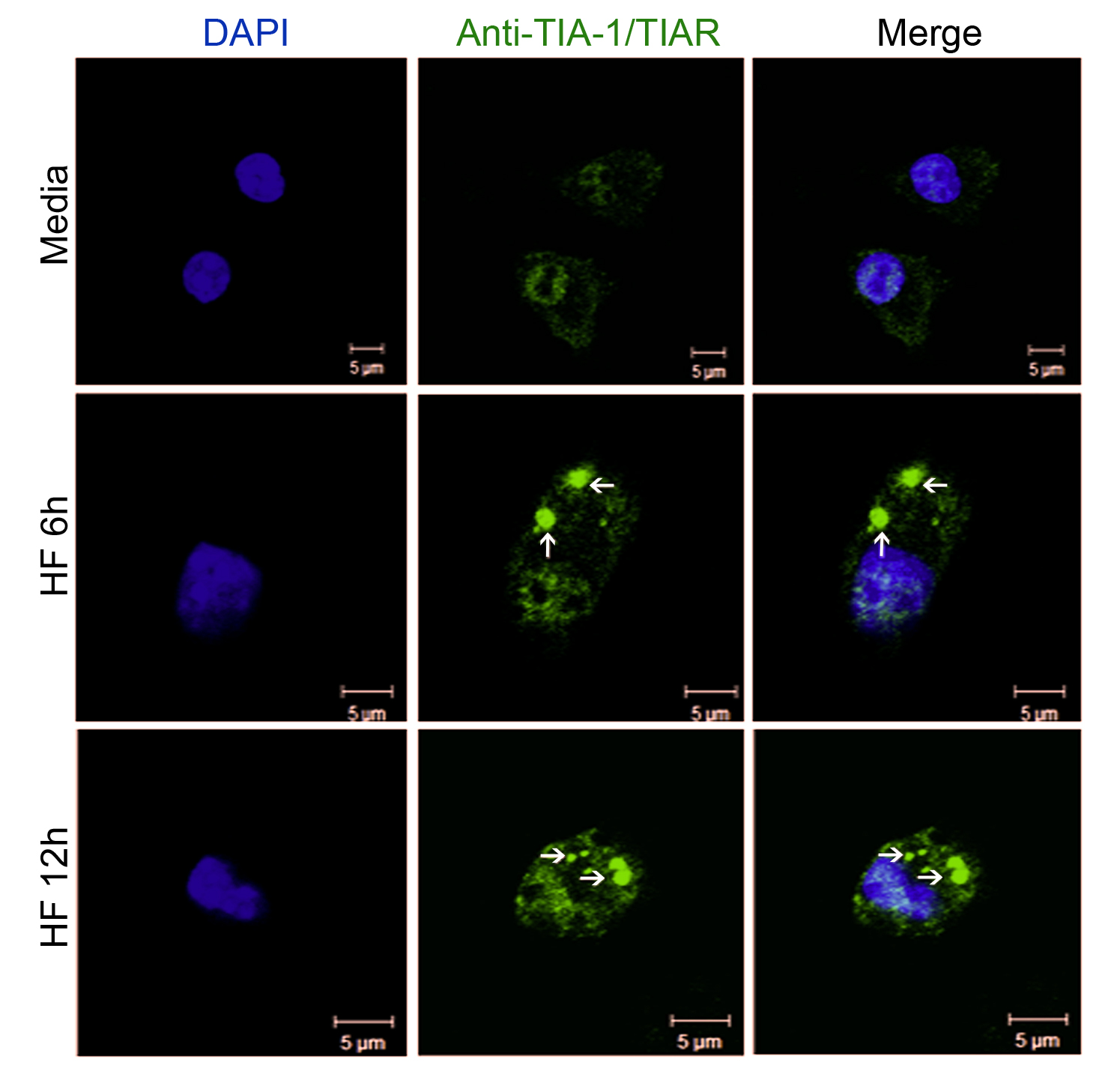

Supplement: S4 Fig — Immunofluorescence imaging of SGs (indicated by white arrows) in BMDMs left untreated (media) or treated with HF (20 nM) at time points specified. Scale bars, 5μm. Data are representative of 1 of 4 independent experiments. BMDM, bone marrow–derived macrophage; HF, Halofuginone; SG, stress granule. (TIF) [file pbio.2005317.s004.tif]

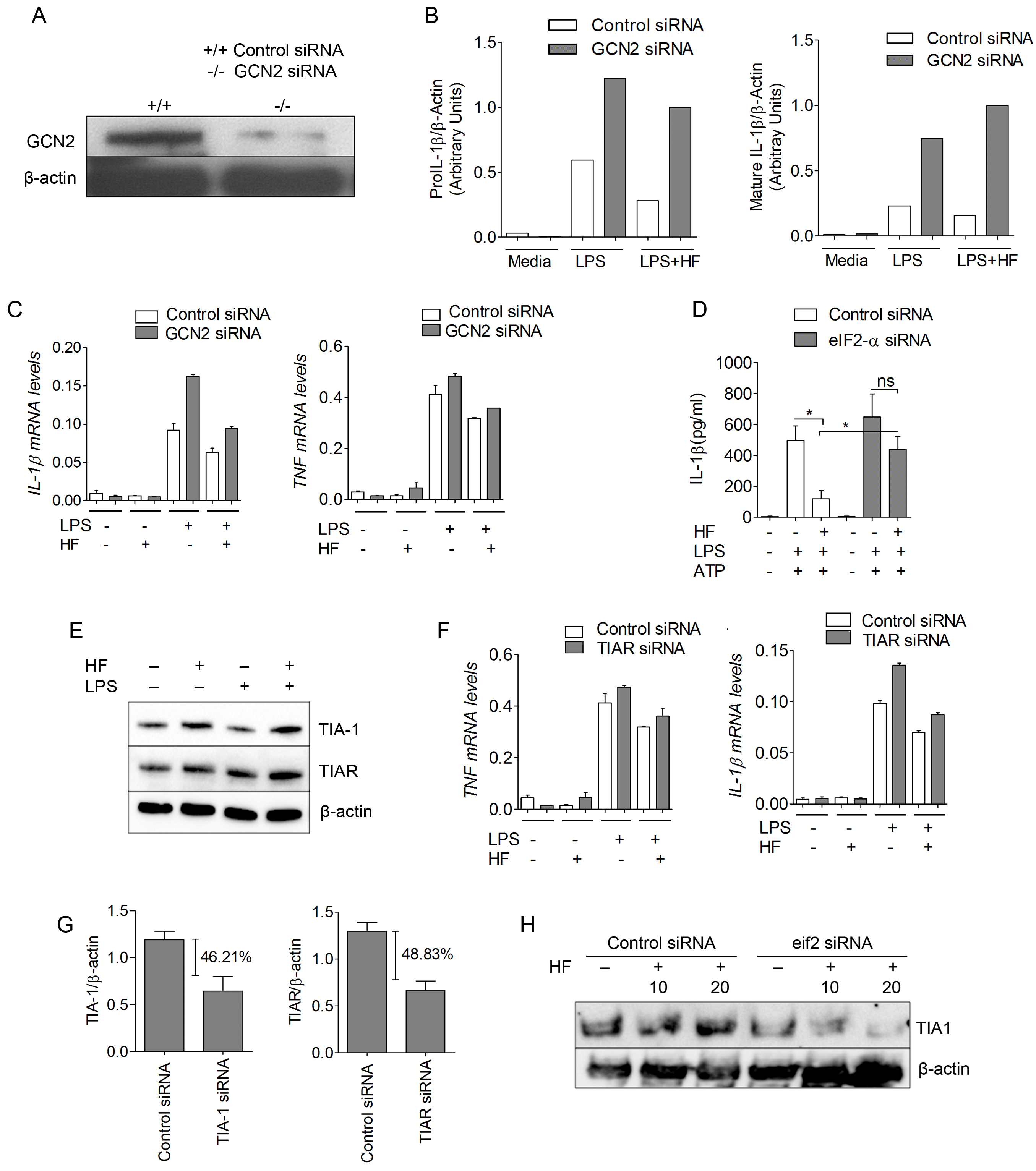

Supplement: S5 Fig — (A) J774A.1 macrophages were transfected with control siRNA- or GCN2-specific siRNA. After 24 h, cell lysates were prepared and analyzed for GCN2 expression by western blotting. (B) Densitometric analysis of western blot (shown in main Fig 2H). The levels of pro and mature IL-1β was normalized to endogenous control β-Actin (S1 Data). (C) qRT-PCR analysis of IL-1β and TNF-α mRNA expression in control or GCN2-silenced macrophages primed with LPS and further treated or untreated with HF (20 nM) (S1 Data). (D) Measurement of IL-1β levels in the culture supernatants of control or eIF2-α siRNA–silenced macrophages treated with LPS or LPS plus HF by ELISA. ATP was added for 30 min at the end of the experiment (S1 Data). (E) Immunoblot analysis of TIA-1/TIAR in HF (20 nM)-, LPS-, or LPS plus HF–treated J774A.1 macrophages. β-actin used as a loading control. (F) qRT-PCR analysis of IL-1β and TNF-α mRNA expression in control or TIAR-silenced J774A.1 macrophages primed with LPS and further treated or untreated with HF (20 nM) (S1 Data). (G) Densitometric analysis of TIA-1 and TIAR expression levels in control siRNA or TIA-1/TIAR siRNA–transfected J774A.1 macrophages. The TIA-1/TIAR levels were normalized to endogenous control β-actin, and percent inhibition in TIA-1/TIAR–silenced macrophages over control was represented in the graph (S1 Data). (H) Immunoblot analysis of TIA-1 in control or eIF2-α–silenced macrophages treated with HF as indicated. β-actin was used as loading control. *P < 0.05. Error bars indicate mean ± SEM. Data represent 1 experiment of 3 independent experiments. eIF2, eukaryotic initiation factor 2; GCN2, general control nonderepressible 2 kinase; HF, Halofuginone; IL-1β, interleukin 1β; LPS, lipopolysaccharide; qRT-PCR, quantitative reverse transcription PCR; RBP, RNA-binding protein; siRNA, small interfering RNA; TIA-1, T cell–restricted intracellular antigen-1; TIAR, TIA-1–related; TNF-α, tumor necrosis factor α. (TIF) [file pbio.2005317.s005.tif]

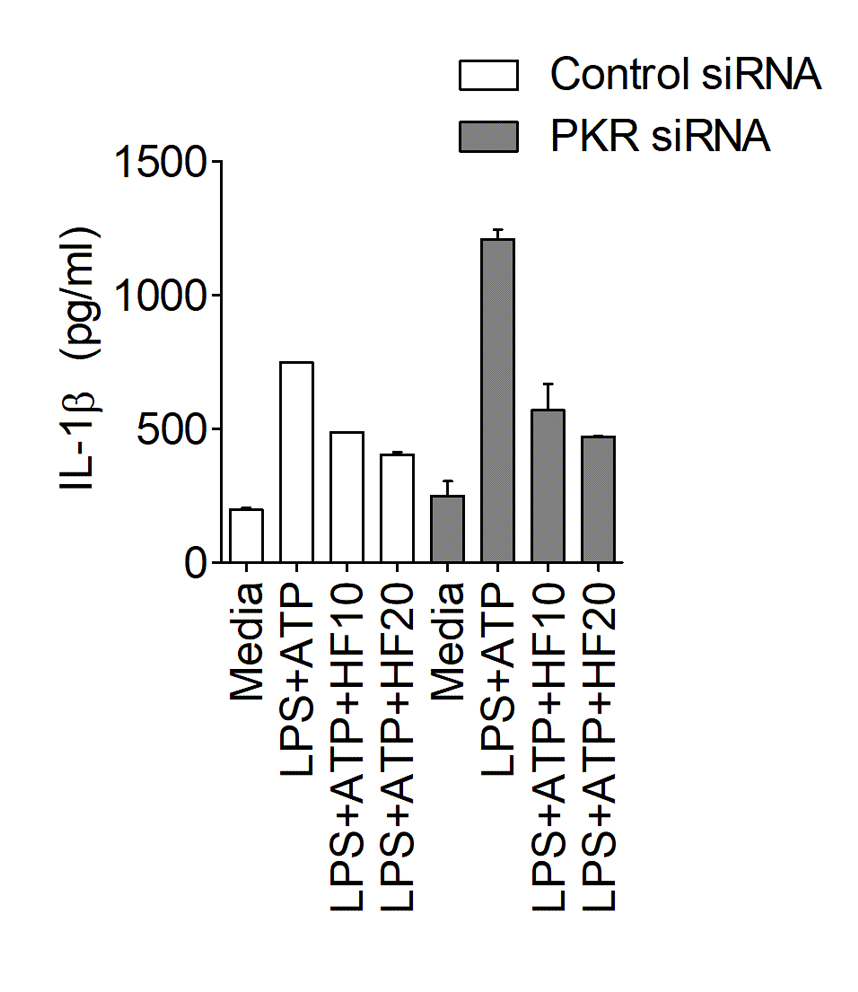

Supplement: S6 Fig — IL-1β protein levels in the culture supernatant of control siRNA–or PKR siRNA–transfected J774A.1 macrophages, stimulated with LPS (500 ng/ml) followed by stimulation with different concentrations of HF. ATP (5 mM) was added to the cultures for 30 min at the end of the experiment (S1 Data). HF, Halofuginone; IL-1β, interleukin 1β; LPS, lipopolysaccharide; PKR, protein kinase R; siRNA, small interfering RNA. (TIF) [file pbio.2005317.s006.tif]

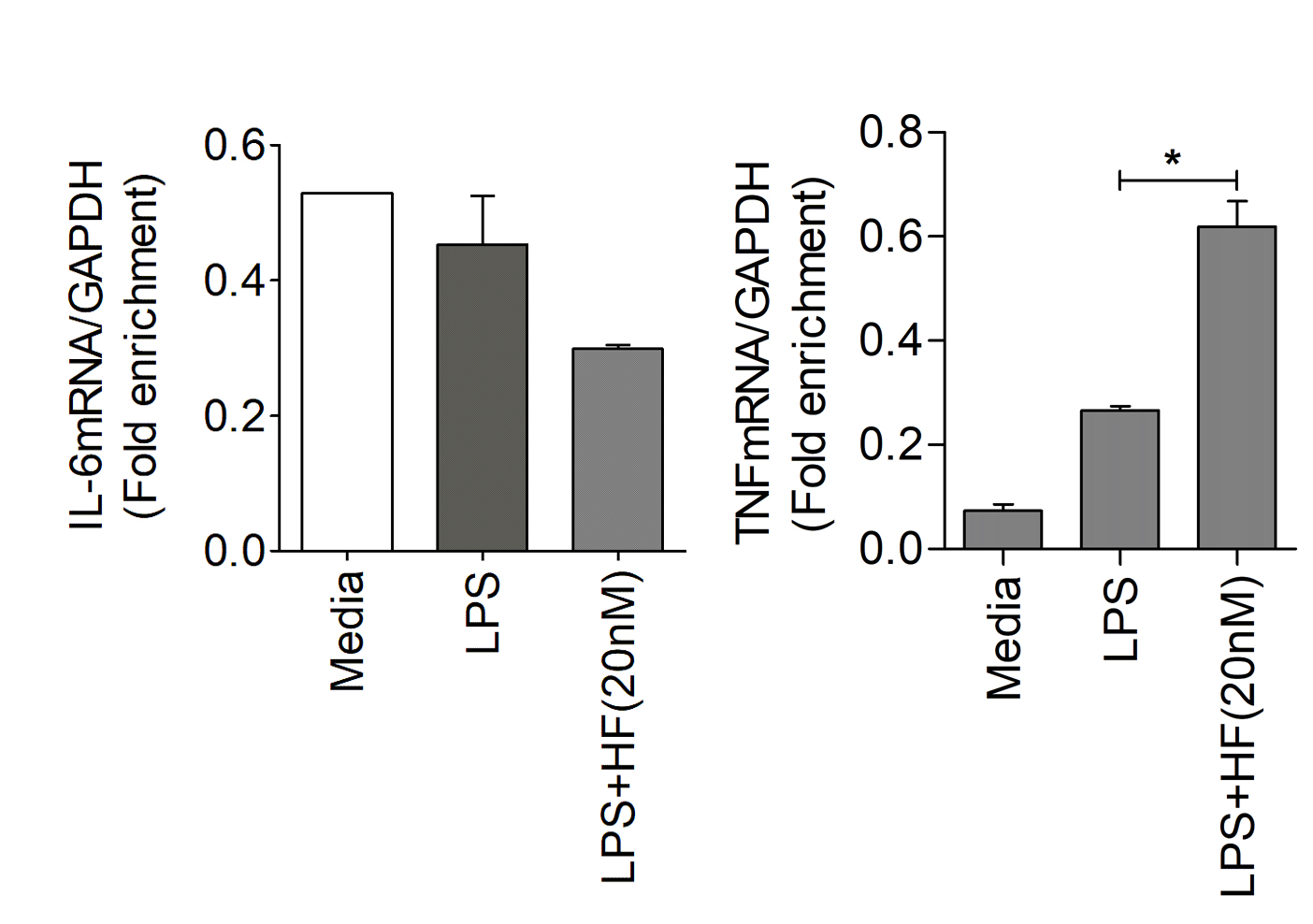

Supplement: S7 Fig — qRT-PCR analysis of IL-6 and TNF-α mRNA in RIP (pull-down using TIA-1/TIAR or IgG) of LPS-primed J774A.1 macrophages untreated or treated with HF (20 nM) (S1 Data). *P < 0.05. Error bars indicate mean ± SEM. Data represent 1 experiment of 3 independent experiments. HF, Halofuginone; IgG, immunoglobulin G; IL-6, interleukin 6; LPS, lipopolysaccharide; qRT-PCR, quantitative reverse transcription PCR; RBP, RNA-binding protein; TIA-1, T cell–restricted intracellular antigen-1; TIAR, TIA-1–related; TNF-α, tumor necrosis factor α. (TIF) [file pbio.2005317.s007.tif]

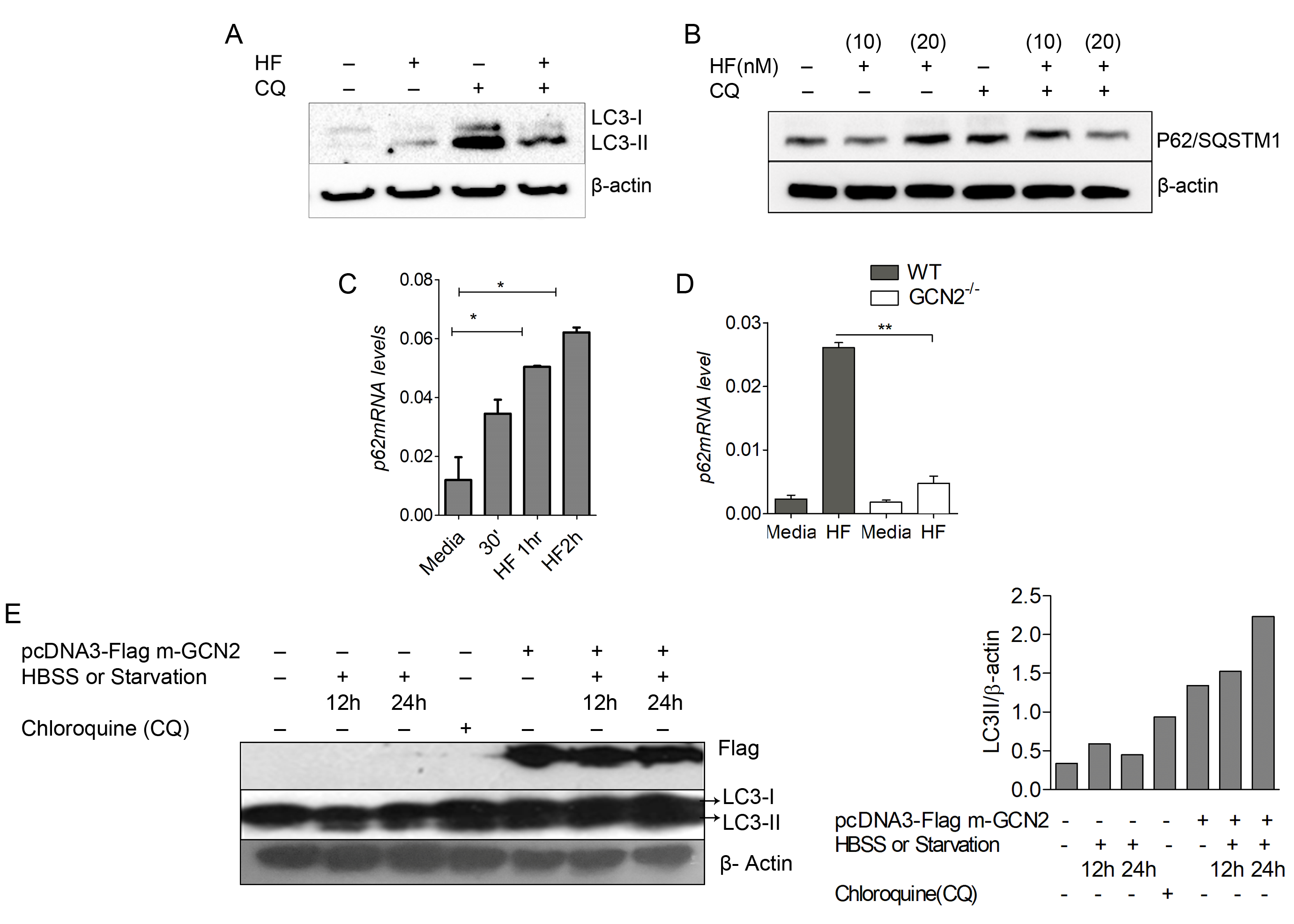

Supplement: S8 Fig — (A) LPS-primed or -unprimed J774A.1 macrophages were treated with HF for 6 h in presence or absence of autophagy inhibitor CQ; lysates were assayed for the expression of autophagy proteins LC3-I and LC3-II by immunoblotting. β-actin was used as loading control. (B) p62 expression in macrophages treated with HF in presence of lysosomal inhibitor, CQ. β-actin was used as loading control. (C) qRT-PCR analysis of p62 mRNA in macrophages treated with HF as indicated (S1 Data). (D) qRT-PCR analysis of p62 mRNA in WT or GCN2−/−macrophages treated with HF (S1 Data). (E) HEK293T cells were transiently transfected with vector or GCN2. After 24 h, cells were starved for indicated time points or treated with chloroquine. Lysates were analyzed for LC3 protein conversion by using immunoblotting. β–actin was used as loading control (S1 Data, right panel). GCN2, general control nonderepressible 2 kinase; HEK293T, human embryonic kidney cells 293T; HF, Halofuginone; LC3, microtubule-associated protein 1A/1B light chain 3; qRT-PCR, quantitative reverse transcription PCR; WT, wild-type. (TIF) [file pbio.2005317.s008.tif]

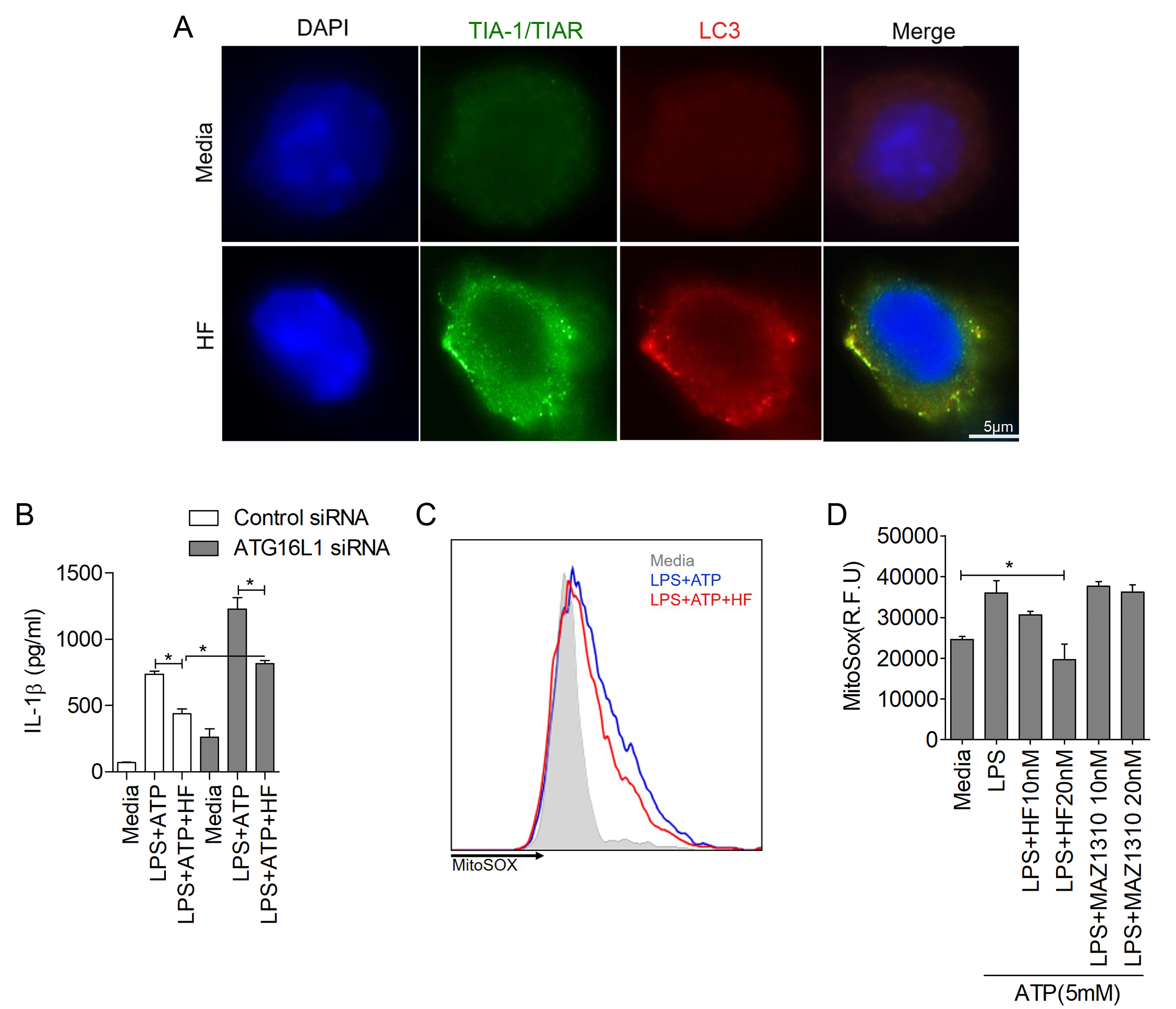

Supplement: S9 Fig — (A) Immunofluorescence microscopy imaging of SGs (stained by TIA-1/TIAR) colocalization with LC3 during HF treatment. Nuclei were stained with DAPI; scale bars, 5 μM. (B) Levels of IL-1β protein in the culture supernatant of control siRNA or Atg16L1 siRNA–transfected J774A.1 macrophages. Macrophages were LPS (500 ng/ml)-primed followed by stimulation with HF (20 nM). ATP (5 mM) was added for 30 min at the end of the experiment (S1 Data). (C, D) Detection of mitochondrial ROS in LPS-primed macrophages stimulated with different concentrations of HF or control MAZ1310 followed by mitochondrial ROS estimation using MitoSOX dye through flow cytometry (panel C), and colorimetric fluorescence measurement (panel D) (S1 Data). *P < 0.05, **P < 0.005. Error bars indicate mean ± SEM. Data represent 1 of 3 independent experiments. HF, Halofuginone; IL-1β, interleukin 1β; LPS, lipopolysaccharide; ROS, reactive oxygen species; SG, stress granule; siRNA, small interfering RNA; TIA-1, T cell–restricted intracellular antigen-1; TIAR, TIA-1–related. (TIF) [file pbio.2005317.s009.tif]

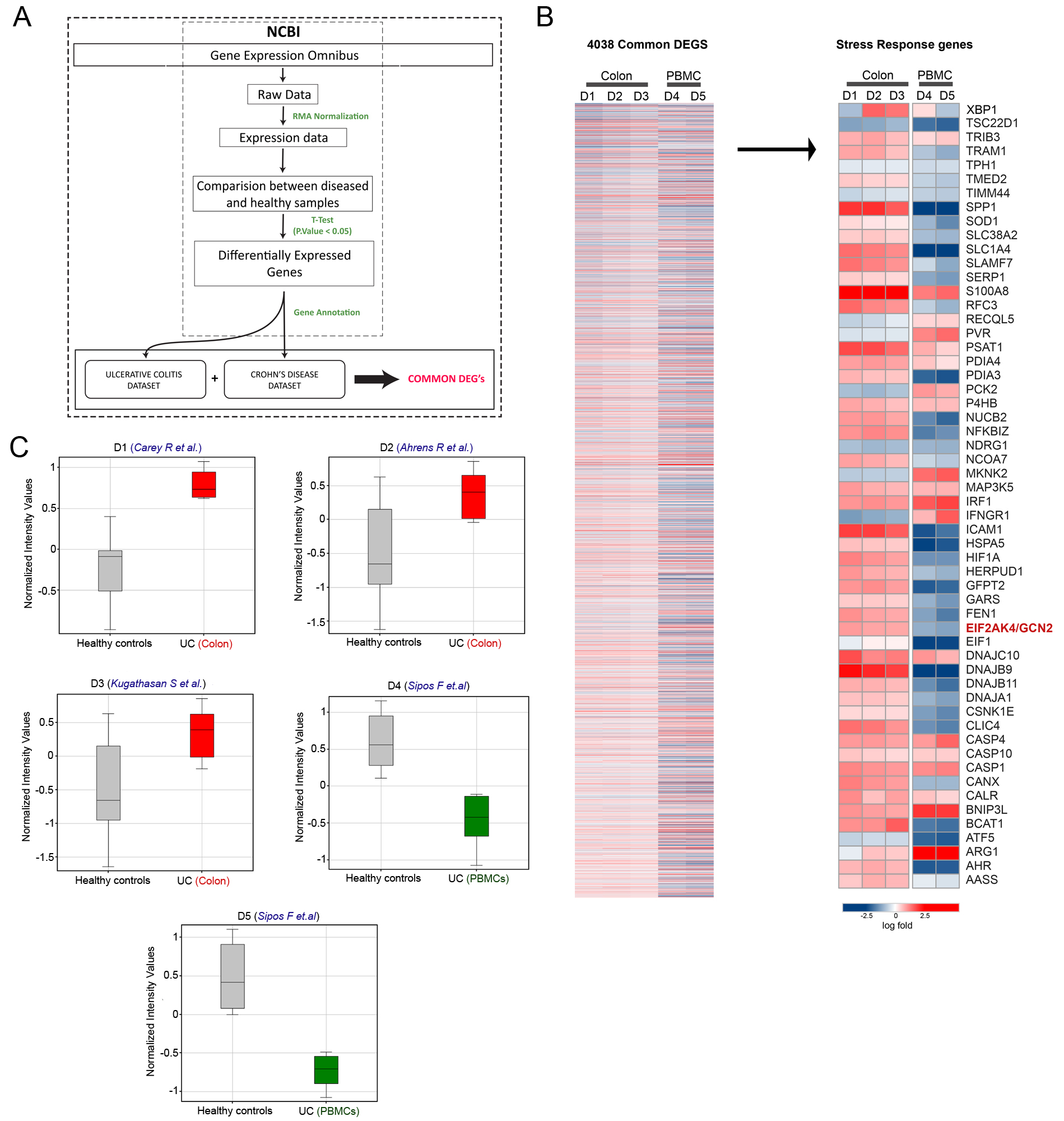

Supplement: S10 Fig — (A) Pipeline of individual gene expression data set analysis followed by meta-analysis. (B) Heat map (left) representing 4,038 DEGs that were commonly expressed across the 5 data sets, in which GCN2 is down-regulated in PBMCs and up-regulated in colon data sets (right heat map). (C) Box-plots representing change in normalized intensity values of GCN2 between diseased (UC and CD) and healthy controls (S1 Data). CD, Crohn’s disease; DEG, differentially expressed gene; GCN2, general control nonderepressible 2 kinase; PBMC, peripheral blood mononuclear cell; UC, ulcerative colitis. (TIF) [file pbio.2005317.s010.tif]
